# Effectiveness of an 11-week exercise intervention for patients with hip or knee osteoarthritis: results of a quasi-experimental pragmatic trial

**DOI:** 10.1186/s13102-023-00779-0

**Published:** 2024-01-20

**Authors:** Inga Krauss, Inka Roesel, Peter Martus, Marco Giurgiu, Gerhard Mueller

**Affiliations:** 1 Department of Sports Medicine, University Hospital and Faculty of Medicine Tuebingen, Hoppe-Seyler-Str. 6, 72076 Tuebingen, Germany; 2 Interfaculty Research Institute for Sports and Physical Activity Tuebingen, Tuebingen, Germany; 3 Institute for Clinical Epidemiology and Applied Biostatistics, University Hospital and Faculty of Medicine, Tuebingen, Germany; 4 Institute of Sports and Sports Science, Karlsruhe Institute of Technology, Karlsruhe, Germany; 5 Allgemeine Ortskrankenkasse AOK Baden-Wuerttemberg, Stuttgart, Germany; 6 Institute for General Practice and Interprofessional Care, University Hospital and Faculty of Medicine, Tuebingen, Germany

**Keywords:** Hip osteoarthritis, Knee osteoarthritis, Group training, Home-exercise, Health services research, Long-term effects, Pain, Function, Health-related quality of life, Joint replacement

## Abstract

**Objective:**

To assess the effectiveness of exercise and education in addition to standard care (SC) compared to SC alone in patients with hip or knee osteoarthritis (OA) during 24 months follow-up.

**Design:**

We conducted a quasi-experimental pragmatic clinical trial in care centers of a health insurance company. Overall, 1,030 subjects with hip and/or knee OA were included. The intervention group was recruited from clients participating in a hip/knee training (HKT, *n* = 515) in addition to SC. The control group (CO, *n* = 515) receiving SC only was recruited from the insurance database. HKT comprised 8 group sessions (1/week) of exercise and education, complemented by a 11-week structured home-exercise program (2/week). Primary endpoints were change of joint-related pain and function (WOMAC Index, score 0–10) after 3 months. Secondary endpoints related to follow-ups at 6, 12 and 24 months. All patient reported outcome measures were analyzed using linear mixed models (LMMs) investigating a time x treatment effect. A multivariable cox proportional hazards regression model was used to identify differences of joint replacement during follow-up between groups.

**Results:**

LMMs revealed statistically significant differences in favor of HKT for the primary outcomes WOMAC pain = 0.47 (CI 0.27–0.66; Effect Size (ES) = 0.22, *p* < 0.001) and WOMAC function = 0.27 (CI 0.11–0.44; ES = 0.13, *p* < 0.001). HKT was superior to CO for 6, 12, and 24 months as well (ES < 0.2, *p* ≤ 0.006). HKT was inferior regarding the first incidence of hip or knee AJR during follow-up in comparison to CO (adjusted hazard ratio, HR = 1.57; CI 1.08—2.30; *p* = 0.020).

**Conclusions:**

This trial demonstrated short-, mid- and long-term superiority of exercise versus control. However, differences were smaller than those reported in previous efficacy trials, raising questions regarding clinical importance. Responder analysis will follow to identify possible predictors for patient responsiveness on an individual level. Further studies should investigate the frequency and reasons for joint replacement following exercise therapy.

**Trial registration:**

German Clinical Trial Register (DRKS00009251). Registered 10 September 2015.

**Supplementary Information:**

The online version contains supplementary material available at 10.1186/s13102-023-00779-0.

## Background

Medical guidelines recommend exercise therapy as a core treatment to alleviate hip and knee osteoarthritis (OA) symptoms [1, 2]. However, there is a considerable discrepancy towards its implementation in healthcare. In 2016, less than 40% of patients with hip, knee or polyarticular OA being customers of a German statutory health insurance company received a prescription for therapeutic exercise [3], and similar numbers have been described in an international meta-analysis on pass rates for the recommendation to exercise in OA care [4]. These numbers highlight the room to improve community-based care [4]. In Germany, statutory health insurance companies can counter undersupply through targeted advice on, and providence of therapeutic exercises for specific patient groups.

Reasons for including therapeutic exercise into clinical recommendations to counteract OA symptoms refer to their effectiveness and safety for patients [1]. Guidelines are primarily derived from expert consensus which is based on an objective review of high-quality meta-analytic results of randomized controlled trials (RCTs) [1]. Prior RCTs report small to moderate effect sizes up to six months after ceasing monitored treatment, yet evidence is limited for long-term benefits [5, 6].

Generalizability of findings from RCTs to real-world populations can be restricted by overestimating effectiveness because of their ideal, controlled conditions [7, 8]. In addition, the above mentioned RCTs compared exercise (intervention group) with non-exercise (control) whereas comparators in pragmatic trials in real-life do not exclude exercise as part of standard care which may decrease superiority of the intervention group because of exercise-related concomitant care of the control. It is therefore of utmost importance to conduct well-designed and carefully described pragmatic trials to evaluate if systematic exercise interventions are advantageous to traditional care [9]. Several countries have implemented community-based exercise and education programs specifically designed for patients with hip and/or knee OA, including but not limited to *Active with OsteoArthritis (AktivA), Better life with Osteoarthritis (BOA)*, *Evidence-based complex intervention for knee and hip osteoarthritis (ESCAPE-pain)* or *Good Life with Osteoarthritis in Denmark (GLA:D®) * [10–13]. For all of the latter, registries for participants have been set up. However, analysis of registry data faces methodological constraints such as the lack of a control group and analysis routines based on all available data only [10–13]. This pragmatic controlled trial therefore aimed to evaluate a scaled-up intervention that was developed on base of an exercise intervention that has previously been shown to be efficious in patients with hip OA [14].

In this study with 24 months follow-up after baseline, we aimed to evaluate whether supplementing standard care (SC) with an efficacious group exercise intervention is more effective than SC alone in patients with hip or knee osteoarthritis. Measures for effectiveness were related to patient-reported pain and physical function (primary endpoints), health related quality of life, general self-efficacy, health-oriented activity status, and risk for artificial joint replacement.

## Methods

### Study design

This 24-months analysis is a quasi-experimental multi-center non-randomized controlled trial compliant to the Declaration of Helsinki, the CONSORT Statement for Randomized Trials of Nonpharmacologic Treatments [15] and the Consensus on exercise reporting template (CERT) [16]. Detailed information on the study design is available in the protocol published by Krauss et al*. * [17]. Important changes to methods after trial commencement are outlined in Additional Information S1.

### Settings and participants

#### Intervention group – Hip and Knee Training (HKT)

Adult customers of the health insurance company Allgemeine Ortskrankenkasse Baden-Wuerttemberg (AOK-BW) with a lifetime prevalence of knee or hip OA and a medical referral to the AOK hip and knee training program were recruited for the intervention group of the present study. The training program was provided at health care centers of the AOK-BW. Subscribers to the training program were asked to sign up for the accompanying scientific evaluation by the exercise instructors and further received a postal mail with a cover letter describing the aim of the accompanying study. This letter explicitly mentioned that persons can contact the principle investigator (PI) of the AOK-BW in case of any questions. The postal mail further included the study information sheet including contact data of both PIs (AOK-BW and University Hospital), a sheet to confirm consent to study participation, the in- and exclusion criteria for study participation and the questionnaires of the outcome measures. Participants were informed that they give consent to study participation by returning the consent sheet and the questionnaire by postal mail.

The main eligibility criteria for participation were (1) prior diagnosis of hip and/or knee OA, (2) AOK-BW health insurance membership for two or more years, (3) absence of any comorbidities which may put the patient at risk while exercising. All in- and exclusion criteria are outlined in Additional Table S2. Returned questionnaires were checked for in- and exclusion criteria. Eligible subjects received further mailings at three (t3), six (t6), twelve (t12) and 24 (t24) months follow-up (FU).

#### Control group (CO)

The database of all insured persons of the AOK-BW was used to recruit participants for CO. They were selected in a two-step process. First, an oversampling of customers for each participant of HKT was chosen according to pre-defined criteria derived from the insurance data base (i.e. osteoarthritis yes/no, co-morbidity, age, gender, joint replacement in the last two years, health care costs etc.). These eligible customers received the same postal information as eligible persons for HKT with the only difference that the cover letter informed about the fact that the AOK-BW needs to recruit patients for the scientific evaluation who do not participate in the AOK hip and knee training. Inclusion and follow-up assessments were identical with HKT. The final statistical twin (1:1 matching) was selected using Propensity Score Matching (PSM). More details on the procedure are outlined in chapter Statistical analyses and in the study protocol [17].

### Interventions

#### Hip and Knee Training (HKT)

The HKT training program was developed based on a previously evaluated 12-week exercise program specifically designed for patients with hip OA [14, 18, 19]. It was complemented by exercises for patients with knee OA and reduced to 11 weeks, consisting of 8 supervised group sessions (1x/week, 60–90 min) and a home-based exercise program (2x/week for 11 weeks) for organizational reasons. All participants of one group started at the same time. Exercises were related to mobilization and motor learning, stretching, strengthening of the lower extremity and postural control. The HKT program was divided into three phases (Table 1).

**Table 1:** Exercise progression of the Hip and knee training (HKT)

Phase	Week	Home-based Training (2/week)	Group sessions Theory/Training (1/week)	Objective
1	1–3	✓	60 min/30 min 40 min/50 min 30 min/60 min	Mobilization Motor learning
2	4–7	✓	30 min/60 min /60 min /60 min /60 min	Balance training (static conditions) Muscular endurance
3	8	✓	- /60 min	Balance training (dynamic conditions) Muscular endurance & strength
	9–11	✓	- / -	

Modified version of the original source: Krauss et al. BMC Public Health 16 (2016) [17]

Exercise progression was defined by dosing specifications for strengthening and balance tasks over the course of the program (Table 2).

**Table 2:** Exercise dosage of the Hip and Knee Training (HKT)

Objective	Sets/Repetitions(reps)/Intensity	Rest
Motor learning	1 set of 10 reps at < 30% MVC	a few sec
Mobilization	1 set of 30 reps at < 30% MVC	< 1 min
Stretching	2 sets of 20 s	a few sec
Muscular endurance	2 sets of 20–25 reps at 30–40% MVC 3 sets of 20–25 reps at 30–40% MVC	1 min 1 min
Strength	3 sets of 10–15 reps at 70% MVC 4 sets of 10–15 reps at 70% MVC	1–2 min 1–2 min
Postural control (static)	1 set of 6 reps of 15 s	30–60 s
Postural control (dynamic)	1 set of 6 reps of 15 s	30–60 s

*MVC* Maximum voluntary contraction. Modified version of the original source: Krauss et al. BMC Public Health 16 (2016) [17]

Participants monitored the intensity of the strengthening exercises through perceived exertion. They were asked to exercise at an intensity that still allowed a correct execution of the last repetition of a given set while rating the perceived exertion as “strenuous” or “very strenuous”. The difficulty of balance tasks should be selected as to be challenging yet executable without compensating movements. For this purpose, participants could choose from several levels of difficulty. Exercise instructors were encouraged to guide dosing during the group sessions accordingly (see below for the training of the exercise instructors).

Individual tailoring of HKT referred to specify exercises for hip or knee osteoarthritis, and the possibility to choose from exercise variations.

Besides physical training, the first four group sessions covered information on exercise related anatomy, joint loading, and dosage. Training materials involved small training devices (i.e. elastic bands, ankle weights) and a book for every participant including general information on hip and knee OA, information on how to dose exercises regarding correct movement execution, perceived exertion and pain, and the structured exercise program for every home-based session of the 11-week training program with a training log. For further details refer to the study protocol [17], the description of exercises (Additional Tables S3 and S4) and the excerpt of the German-language exercise book [20].

Group sessions with a maximum group size of twelve participants were supervised by health care professionals of the AOK-BW who had been trained by the developers of the intervention program (University Hospital of Tuebingen, Dept. of Sportsmedicine). Supervisors received the exercise book and a comprehensive exercise instructor manual including presentations to guide the educational elements of the group sessions. Exercises for the home-based training were introduced in the group sessions. Treatment fidelity of care providers to the protocol during the study period was not specifically enhanced and not monitored. HKT was provided on top of the regular utilization of standard care that was provided or prescribed by patients’ physicians.

#### Control group (CO)

The control group received all services that were regularly provided or prescribed by the patients' physicians and therefore corresponded to the real-life scenario of patient care in OA (= standard care, SC). SC could consider any form of medical care (i. e. medication, physiotherapy, referral to exercise, orthotics, joint replacement etc.).

## Measures

Outcome measures for CO and HKT were assessed at baseline (t0) and after three (t3), six (t6), twelve (t12), and 24 (t24) months using self-administered questionnaires which were delivered with a return envelope by postal mail. Economic data and ICD-Codes (International Classification of Diseases) for knee and hip arthroplasty were assessed from the insurance data base. Economic data were used for the propensity score matching (see section *Statistical analyses*).

### Patient baseline characteristics (t0 only)

Self-reported patient characteristics comprised age, sex, body mass index (BMI), site of OA (hip/knee/both), additional joint replacement (yes/no). The following data were obtained from the insurance data base: working status, complexity of work, years of school education and level of education.

### Primary outcomes (t0 – t3)

#### WOMAC pain and function

The subscales *pain* and *physical function* of the Western Ontario and McMaster Universities Osteoarthritis Index (WOMAC® NRS 3.1 German Index) were used as primary outcomes. The scales in this study ranged from 0 (no limitation) to 10 (maximum limitation).

### Secondary outcomes (t0—t24)

#### WOMAC pain and function

WOMAC follow-up data t6—t24 were used to assess mid- and long-term effects of the intervention.

#### Health-related quality of life (VR-12, PCS, MCS)

The Veterans RAND 12-Item Health Survey (VR-12) is a patient-reported global health measure that assesses a patient’s overall perspective of their health [21]. The instrument comprises 12 items, and the questions correspond to eight different health domains: *general health perceptions (GHP), physical functioning, role limitations due to physical and emotional problems, bodily pain, energy-fatigue levels, social functioning, and mental health.* The VR-12 uses five-point ordinal response choices (1 = *no, none of the time* to 5 = *yes, all of the time;* higher scores represent better health status). Answers were summarized in a Physical Component Score (PCS) and a Mental Component Score (MCS), each normalized to the 1990 US population norm (mean = 50; SD = 10).

#### General self-efficacy scale (GSE)

The GSE scale is a ten-item self-report psychometric scale that measures general self-efficacy as a prospective and operative construct [22]. Items are scored on a 4-point Likert scale (1 = *not at all true* to 4 = *completely true,* higher scores indicate higher self-efficacy). A mean score was calculated when at least six items were present.

#### Health-oriented activity status (Ho-AS)

Participants were asked to rate whether they are active in a health-oriented manner (Ho-AS), e.g., visiting gyms, going for a run or walk (1 = *outstandingly active* to 5 = *not at all active*).

#### Artificial joint replacement during follow-up (t3 – t24)

First incidence of artificial joint replacement (AJR) at the knee or hip joints during follow-up t3—t24 was read out from routine data of the insurance data base.

#### Perceived benefit from the intervention/satisfaction with exercise instructors (t3, HKT only)

The participants’ overall perceived benefit from the intervention was assessed on a 5-point Likert scale (1 = *very high perceived benefit* to 5 = *no perceived benefit*). Furthermore, questions on trainer competence (1 = *very competent* to 4 = *not competent at all*), trainer motivation (1 = *very engaged and motivated* to 4 = *not engaged and motivated at all*) and whether participants would recommend the training program to others (1 = *definitely yes* to 4 = *definitely not*) were asked.

#### Exercise adherence (t3, HKT only)

Participants of HKT were asked to report if they attended all group sessions (yes/no), all home-based exercise sessions (yes/no) and reasons for non-participation (multiple responses possible), if applicable.

#### Exercise-related adverse events (t3, HKT only)

Occurrence of exercise-related pain and its frequency, duration and intensity were collected.

#### Concomitant care (t3 – t24)

Participants of CO (t3—t24) and HKT (t6 – t24) were asked to report participation in a hip and/or knee training during the previous follow-up period. Programs were differentiated into HKT group training and HKT home-based training, AOK machine-based training (another specific offer of the AOK-BW, specifically designed for patients with hip/knee OA) or any other exercise training for hip/knee OA (provider not specified). Participants were further asked if they attended any other additional AOK-provided health care offers.

### Sample size

The sample size was estimated on the empirical basis of a previous RCT [17]. In this RCT intra-individual differences of the WOMAC pain subscale and as well the WOMAC physical function subscale exhibited an effect size according to Cohen’s d of 0.5 between intervention and control group. Based on these results and a potential efficacy-effectiveness gap between RCTs and studies under real life conditions [23] we finally assumed an effect size of ES = 0.3. Accounting for the two primary endpoints (WOMAC pain, physical function), a level of significance of 0.025 (two-sided, Bonferroni correction) and a power of 0.90 was used. Calculations yielded a sample size of 278 subjects per group in a parallel group design (nQuery 7.0). Accounting for a dropout rate of 20% (*n* = 350 subjects/study arm) and cluster effects of subjects within treatment groups, *n* = 700 participants should be allocated to each treatment arm. Further details are provided in the study protocol [17] and Additional Information S1.

### Blinding

Blinding of the subjects or care providers to treatment was not possible as treatment exposure was evident. Blinding of assessors was not applicable as all outcomes were patient reported or retrieved from the health insurance data base. Statisticians were not blinded due to the necessary preparation of the baseline data of the intervention group for PSM.

### Statistical analyses

All data analyses were conducted with SPSS Statistics version 26 (IBM Corp. Armonk, N.Y., USA) and R version 4.0.4 (R Core Team, 2020) with R Studio (version 1.3.1056; RStudio, PBC., Boston, MA, USA).

### Matching procedures for the control group

**Fig. 1:**
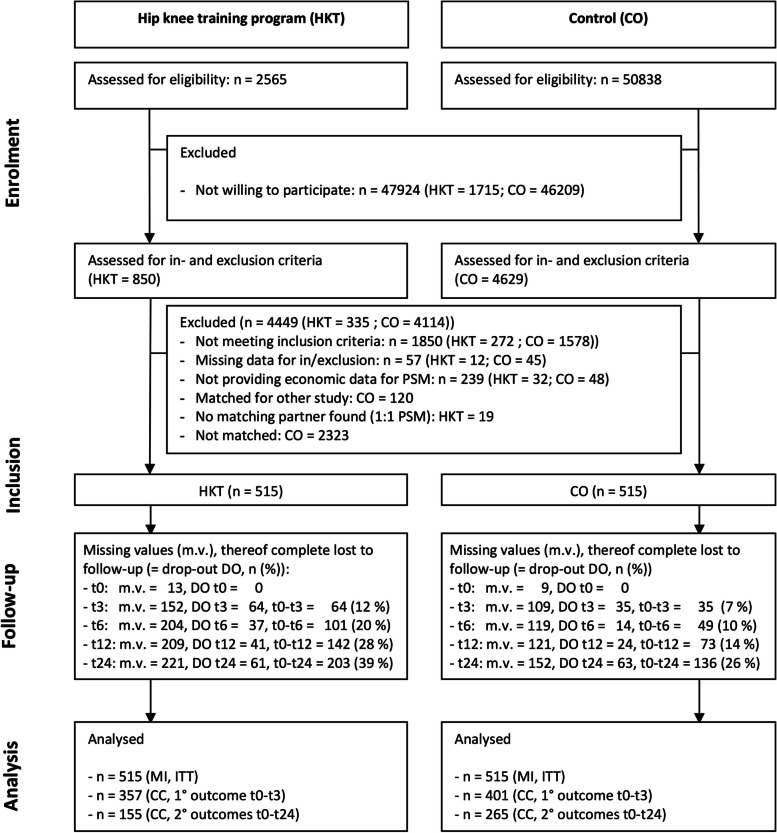
Flow diagram

The matching procedure for the statistical twins of CO to each participant of HKT was conducted in two steps. First, customers of the AOK-BW were assessed for eligibility from the insurance data base according to pre-defined matching criteria (Additional Table S5). This step was done quarterly after including new subjects into HKT. We aimed to recruit ten customers of the AOK-BW for participation in the control group (CO) for each participant of HKT. Due to the low response rate, however, around 60 insured persons per HKT participant had to be selected and contacted in order to have a ratio of 1:4 for the final matching (see Fig. 1). Socio-demographic (age, sex), health-related (BMI, OA-related pain and function, affected joint, previous artificial joint replacement physical and mental health-related quality of life, QALY, health-related activity, general self-efficacy), and economic variables (unspecific and specific health care costs and days of disability) were included in the final matching. The standardized mean difference (SMD) for all covariates was < 9% (see Additional Table S 5).

### Imputation of missing data

To investigate the mechanism of missing data, we performed Little’s test [24], which yielded a statistically significant result (p < 0.001), so the null hypothesis of missing completely at random (MCAR) was rejected. As missingness was mostly due to wave-nonresponse with patients being lost to follow-up, we further explored a missing at random (MAR) mechanism by comparing the characteristics of dropouts vs. completers of the study (see results section). Multiple imputation (MI) was then performed with the R package *Amelia* [25] under the assumption that data are missing at random (MAR). A two-step MI procedure [26] was chosen to combine the selection of statistical twins from the control group via PSM, which was based on imputed baseline data (t0) only, and the multiple imputation of the longitudinal follow-up data with the final matched pairs (t3, t6, t12, t24). M = 100 MI sets were generated in total.

### Main analysis

Two separate linear mixed models (LMMs) for the primary endpoints WOMAC pain and function were conducted with a restricted maximum likelihood estimation (REML) including *time* (t0, t3) and *treatment* (HKT, CO) and *time* x *treatment* interaction as fixed factors with a random intercept for subject to account for within-subject correlations. We refrained from analyzing our data using a matched-pair design, as PSM does not guarantee individual pairs to be well-matched on the full set of covariates and included the PS as a covariate in the models instead [17]. Model assumptions were checked visually by means of residual- and QQ-plots (normality of residuals, normality of random effects, linearity, homogeneity of variance). Logarithmic transformations were applied to both primary outcomes to achieve normal distribution. Overall omnibus F-tests (pooled over the MI sets) were conducted to check for statistically significant *time x treatment* effects. To interpret the magnitude of the treatment and time effects, pooled estimated marginal means (EMM) and the corresponding 95% confidence interval (CI) were calculated and back-transformed from log-scale to the original measurement scale. From those EMMs, within-group change from baseline (cfb) estimates and the according estimated between-group treatment differences (ETD) were derived for each timepoint. Similar LMMs were run for long-term follow-ups (t0-t24) for all secondary outcomes including WOMAC pain and function (both with logarithmic transformation), GSE, MCS, PCS, and Ho-AS. Effect sizes (ES) were calculated using the estimates derived from the LMM analyses. Estimates were divided by the pooled SD of HKT and CO at baseline. Effect sizes were considered to be small (0.2–0.29), moderate (0.3–0.79) or large (> 0.8) [27].

Statistical significance for the two primary outcomes was set as p ≤ 0.025 (two-sided, Bonferroni correction). For secondary outcomes, statistical significance was set as p ≤ 0.05 without claiming confirmatory interpretation.

### Additional analyses

#### Sensitivity analysis (pre-specified in the study protocol)

We ran the LMMs for WOMAC pain and WOMAC function on all available data (AA) without MI. To further evaluate the robustness of our results we also conducted a complete case (CC) analysis on the two primary endpoints. At this point it is noted that CC dataset has unequal group sizes and does not contain all matched 1:1-pairs.

#### Exploratory subgroup analysis

A subgroup analysis was done to compare WOMAC pain and WOMAC function at t3 versus baseline for complete cases of HKT versus a subsample of CO (CO-exercise). CO-exercise was defined as participants of CO having reported to engage in any hip/knee-specific exercise between t0 and t3 as outlined in Additional Table S 14. Again, it is noted that the subgroup dataset has unequal group sizes and does not align to all matched 1:1-pairs.

#### Exploratory analysis on artificial joint replacement during follow-up (t0 – t24)

An exploratory time-to-event analysis was conducted applying a multivariable cox proportional hazards regression model for the first incidence of joint replacement (AJR) in the follow-up period t0 – t24 to identify risk factors including the covariates intervention group, WOMAC pain, MCS and PCS at baseline (t0) as well as age, sex and site of OA. Variables that were excluded from the model with the respective reasons are outlined in Additional Information S1. Results were reported as hazard ratios (HR), 95% confidence intervals (CI) and two-sided p-values. The proportional hazard (PH) assumption required for Cox proportional hazards modelling was found to be fulfilled by inspecting the respective Schoenfeld residuals and *time* x *covariate* interactions.

## Results

### Participants (Fig. 1)

First and last mails to participants of HKT were sent in September 2015 and April 2019 (first patient in: 22 September 2015). First and last postal mails to participants of CO were sent in February 2016 and September 2019, respectively. The trial was ended before reaching the target sample size of *n* = 700 for HKT and the requested ten-fold number for CO, as response rates to postal mailings were much lower than expected. Compared to the planned time-line in the study protocol time for recruitment was extended by one and a half year, but this could not fully compensate the lower rates [17]

Participants of HKT were recruited from AOK hip and knee training courses taking place from September 2015 to April 2017. In this period, HKT was offered across the federal state of Baden-Württemberg (45 locations in 2015, 73 locations in 2016, and 14 locations in early 2017). From these courses, 2565 customers received the postal mailing for study participation. Participants matched according to pre-defined criteria and assessed for eligibility for the control group (n = 50,838) were selected from the AOK-BW insurance database and invited to study participation by letter as outlined above (first CO-patient in: February 2016). In total 5479 questionnaires were returned (HKT = 850, CO = 4629), of which n = 4449 (HKT = 335, CO = 4114) were excluded. Finally, a statistical twin from the pool of eligible control group participants could be matched for *n* = 515 participants of HKT, thus 1030 subjects were included in our study. For population characteristics before and after the matching process, see Additional Table S5, details on participant flow are outlined in Fig. 1.

### Dropouts

The rate of participants who prematurely dropped out of the study was 32.9% (*n* = 339) overall, 39.4% (*n* = 203) for HKT, and 26.4% (*n* = 136) for CO, respectively. For HKT, female participants were more likely to drop out. For CO, participants suffering from hip and knee OA or hip OA were more likely to drop out. Dropouts exhibited significantly higher baseline WOMAC pain and limited function (overall, HKT, and CO). Dropouts further exhibited a worse physical component (overall and CO) and a worse mental component score (CO) (Additional Table S6).

### Patient baseline characteristics (t0 only)

Baseline characteristics of the study population are displayed in Table 3 and Additional Table S 7. Both, matched and non-matched patient characteristics of the two groups were alike.

**Table 3:** Baseline characteristics of the matched pairs study population (*n* = 1030)

	**Hip and Knee Training (HKT)** *n* = 515	**Control (CO)** *n* = 515
Women (n, %)	393 (76.3)	396 (76.9)
Age (years), mean (SD)	63.55 (9.49)	63.73 (9.01)
Body Mass Index (kg/m2), mean (SD)	27.89 (4.55)	27.74 (4.92)
OA lifetime prevalence, (n, %)		
Knee	264 (51.3)	264 (51.3)
Hip	116 (22.5)	116 (22.5)
Both	135 (26.2)	135 (26.2)
Joint replacement (hip/knee), (n, %)	65 (12.6)	65 (12.6)
WOMAC, Mean (SD), [Median (IQR)]		
Pain	3.14 (1.99), [2.80 (1.60, 4.39)]	3.16 (2.31), [2.80 (1.20, 4.80)]
Function	2.78 (1.93), [2.53 (1.24, 3.88)]	2.74 (2.20), [2.24 (0.94, 4.24)]
VR-12, Mean (SD)		
PCS90	38.36 (8.15)	38.62 (8.74)
MCS90	50.00 (11.19)	49.70 (10.88)
General self-efficacy scale (GSE), Mean (SD)	3.08 (0.55)	3.07 (0.56)
Health-oriented activity status (Ho-AS), Mean (SD)	2.91 (0.87)	2.90 (0.95)

*HKT* Hip and knee training, *CO* Control, *IQR* Interquartile range, *WOMAC* subscales pain and function, with scores ranging from 0 to 10 (best to worst scale). VR-12: Veterans Rand-12 of which the Physical component score (PCS90) and the Mental Component Score (MCS90) were calculated with a value of 50 indicating the mean American Norm 1990 (worst to best). General self-efficacy scale (GSE) with scores ranging from 1–4 (worst to best) and Health-oriented activity status (Ho-AS) with scores ranging from 1 to 5 (best to worst)

### Primary outcomes (t0—t3)

#### WOMAC pain and function

HKT showed superior results for WOMAC pain and function as compared to CO (significant *time x treatment* for WOMAC pain (F(1,1028) = 21.54, Effect size (ES) = 0.22, *p* < 0.001), and WOMAC function (F(1,1028) = 10.54, ES = 0.13, *p* = 0.001, Additional Table S 8, Table 4).

**Table 4:** Within-group estimates of change from baseline (cfb, 95% CI) and the according between-group estimated treatment differences (ETDs) at t3, t6, t12 and t24 months for the primary and secondary outcomes

	**Control (CO)** ***n*** = 515	**Hip Knee Training (HKT)** ***n*** = 515	**Estimated treatment difference (ETD)** **CO—HKT**	***p*-value**	**Effect Size**
*Primary Outcomes*					
WOMAC					
pain t3	0.21 (0.07; 0.35)	-0.25 (-0.39; -0.11)	0.47 (0.27–0.66)	** < 0.001**	0.22
function t3	0.32 (0.21; 0.44)	-0.05 (-0.06; 0.17)	0.27 (0.11–0.44)	** < 0.001**	0.13
*Secondary Outcomes*					
WOMAC					
pain t6	0.15 (-0.07; 0.36)	-0.14 (-0.36; 0.07)	0.29 (0.08; 0.51)	**0.008**	0.13
pain t12	0.11 (-0.11; 0.32)	-0.23 (-0.44; -0.01)	0.33 (0.12; 0.55)	**0.003**	0.15
pain t24	0.20 (-0.01; 0.44)	-0.14 (-0.35; 0.08)	0.34 (0.12; 0.55)	0.**002**	0.16
function t6	0.36 (0.17; 0.55)	0.14 (-0.05; 0.33)	0.22 (0.03; 0.41)	**0.020**	0.11
function t12	0.39 (0.20; 0.58)	0.05 (-0.13; 0.24)	0.33 (0.14; 0.52)	**0.001**	0.16
function t24	0.43 (0.24; 0.62)	0.14 (-0.05, 0.34)	0.29 (0.09; 0.48)	**0.003**	0.14
Veterans Rand-12 (VR-12)					
Physical Component Score t3	-0.05 (-0.96; 0.85)	0.83 (-0.08; 1.73)	-0.88 (-1.80; 0.36)	0.060	0.10
Physical Component Score t6	0.22 (-0.69; 1.12)	0.86 (-0.04; 1.77)	-0.65 (-1.57; 0.27)	0.167	0.08
Physical Component Score t12	-0.02 (-0.92; 0.89)	1.39 (0.49; 2.30)	-1.41 (-2.33; -0.49)	**0.003**	0.17
Physical Component Score t24	0.70 (-0.20; 1.61)	0.87 (-0.04; 1.78)	-0.17 (-1.09; 0.76)	0.726	0.02
Mental Component Score t3	-0.26 (-1.39; 0.87)	0.71 (-0.42; 1.84)	-0.97 (-5.14; -0.52)	0.096	0.09
Mental Component Score t6	-0.12 (-1.24; 1.01)	0.15 (-0.98; 1.29)	-0.27 (-5.00; -0.36)	0.645	0.02
Mental Component Score t12	0.21 (-0.92; 1.34)	-0.37 (-1.50; 0.77)	0.58 (-6.47; -1.83)	0.326	-0.05
Mental Component Score t24	-0.49 (-1.62; 0.64)	-0.53 (-1.66; 0.61)	0.04 (-5.23; -0.60)	0.945	0.00
General self-efficacy scale (GSE)					
GSE t3	-0.00 (-0.06; 0.05)	0.00 (-0.05; 0.06)	-0.00 (-0.06; 0.05)	0.857	0.00
GSE t6	-0.02 (-0.07; 0.04)	0.01 (-0.05; 0.06)	-0.03 (-0.08; 0.03)	0.338	0.05
GSE t12	-0.04 (-0.09; 0.02)	-0.01 (-0.06; 0.05)	-0.03 (-0.08; 0.02)	0.268	0.05
GSE t24	-0.03 (-0.08; 0.03)	0.02 (-0.04; 0.07)	-0.04 (-0.10; 0.01)	0.104	0.07

Linear Mixed Models (Time, Treatment, Time*Treatment, PS Propensity Score), WOMAC (0–10, best to worst): logarithmic estimates back-transformed to original scale, Veterans Rand-12 (VR-12, worst to best) of which the Physical component score (PCS90) and the Mental Component Score (MCS90) were calculated with values of 50 indicating the mean American Norm 1990, General Self-efficacy (GSE,1–4, worst to best). Scales best to worst (WOMAC): positive estimated treatment difference indicate benefit for HKT. Scales worst to best (VR-12, GSE): negative estimated treatment difference indicate benefit for HKT. *P*-values for time*treatment interaction of the LMMs. Significance in bold is set at alpha = .0025 to account for post-hoc testing with respect to the two primary outcomes. For secondary outcomes alpha = 0.05 without claiming confirmatory interpretation of *p*-values. Positive effect sizes indicate benefit for HKT versus CO

#### Secondary*** outcomes (t0—t24)***

**Fig. 2:**
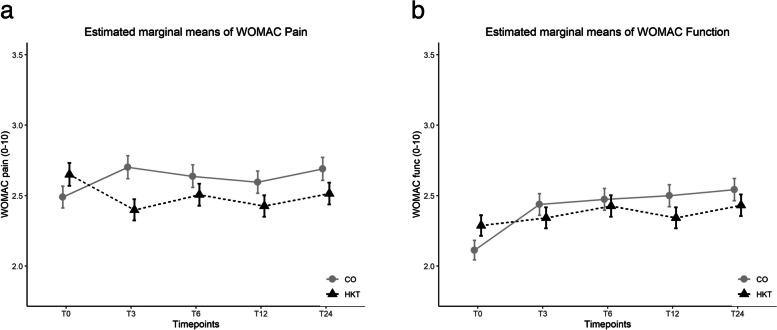
**a** Estimated marginal means (EMM) ± standard error (SE) of WOMAC Pain: 24-months follow-up. **b** Estimated marginal means (EMM) ± standard error (SE) of WOMAC Function: 24-months follow-up. **a**/**b** legend: Hip Knee Training (HKT), Control (CO). Logarithmic EMM were back-transformed to the original scale

HKT showed superior results for WOMAC pain and function as compared to CO (*time* x *treatment* for WOMAC pain t0—t24 (F(4, 4112) = 4.88, *p* < 0.001), and WOMAC function t0—t24 (F(4, 4112) = 3.63, *p* = 0.006)) (Additional Table S 9). ES for all measures were smaller than 0.2 (Table 4 and Fig. 2a/b). HKT also showed superior results for PCS as compared to CO (*time* x *treatment* for PCS t0-t24 (F(4, 4112) = 2.92, p = 0.020)). However, superiority of HKT versus CO was only found for t12 versus baseline (*p* = 0.003, ES = 0.17). There was no statistically significant *time x treatment* for MCS (F(4, 4112) = 1.84, *p* = 0.116) and GSE (F(4, 4112) = 0.91, *p* = 0.46). For Ho-AS, superiority of HKT versus CO was given for all time points t0 – t24 (*time* x *treatment* for Ho-AS t0—t24 (F(4, 4112) = 6.40, *p* < 0.001) with ES between 0.24 and 0.31). Estimated marginal means (EMM) for all secondary outcomes are outlined in Table 4 and 5.

**Table 5:** Primary and secondary outcomes at t3, t6, t12 and t24 months change from baseline (cfb, 95% CI) in the intention-to-treat population

	**Control (CO)** ***n*** = 515	**Hip Knee Training (HKT)** ***n*** = 515	**Estimated treatment difference CO—HKT**	***p***-value	**Effect Size**
*Secondary Outcomes (cont.)*					
Health-oriented activity status (Ho-AS)					
Ho-AS t3	0.15 (0.03; 0.27)	-0.10 (-0.22; 0.03)	0.25 (0.13;0.37)	** < 0.001**	0.27
Ho-AS t6	0.18 (0.06; 0.31)	-0.04 (-0.17; 0.09)	0.23 (0.10; 0.35)	** < 0.001**	0.25
Ho-AS t12	0.19 (0.07; 0.31)	-0.03 (-0.16; 0.09)	0.22 (0.10; 0.35)	** < 0.001**	0.24
Ho-AS t24	0.23 (0.11; 0.36)	-0.05 (-0.17; 0.08)	0.28 (0.15; 0.41)	** < 0.001**	0.31

Linear Mixed Models (Time, Treatment, Time*Treatment, *PS *Propensity Score), Health-oriented activity status (Ho-AS, 1-5, best to worst). Scales best to worst (Ho-AS): positive estimated treatment difference indicate benefit for HKT. *P*-values for time*treatment interaction of the LMMs. For secondary outcomes alpha = 0.05 without claiming confirmatory interpretation of *p*-values. Positive effect sizes indicate benefit for HKT versus CO

#### Sensitivity analyses for WOMAC pain and function

The sensitivity analyses for the primary endpoint t3 versus baseline and for FU t0—t24 on all available data (AA) without MI as well as on the complete case (CC) dataset showed that findings are robust and consistent with results from our primary analyses (Additional Table S9). However, absolute differences and effect sizes in the mid- and long-term were larger for AA and CC analyses (Additional Tables S10 and S11) which might indicate a bias ignoring the pattern of missing values.

#### Exploratory subgroup analyses for WOMAC pain and function (HKT versus CO-exercise, t0—t3)

Baseline data of the complete case subgroup analyses of HKT (*n* = 357) in comparison to CO-exercise (*n* = 178) only differed for the health-oriented activity status with participants of CO-exercise being more active in comparison to HKT (*p* < 0.001) (Additional Table S16). However, results of the subgroup analyses for WOMAC pain and function t3 versus baseline were consistent with results of the primary analysis as well as the sensitivity analyses for AA and CC (Additional Table S17).

#### Artificial joint replacement during follow-up (t0—t24)

**Fig. 3:**
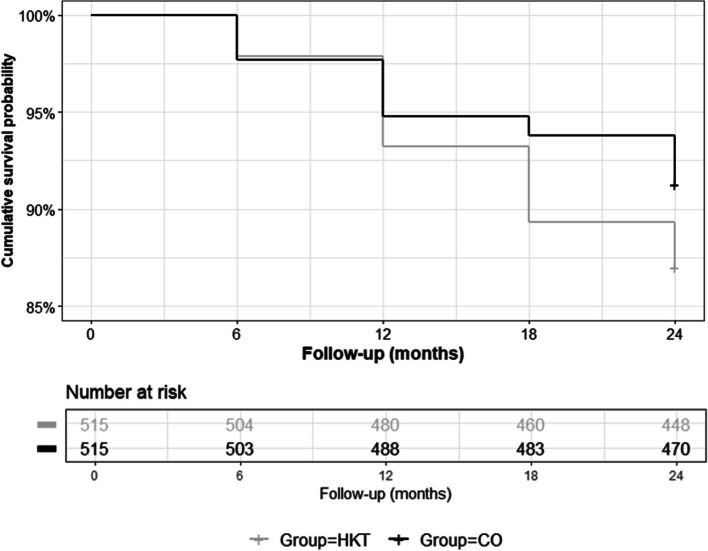
Adjusted cox regression model: cumulative survival probability

HKT was inferior regarding the first incidence of hip or knee AJR during FU in comparison to CO with 67 (13%) versus 45 (8.7%) events. After adjustment for age, sex, site of OA, and baseline scores for MCS, PCS and WOMAC pain a significant difference in time to AJR (Hazard ratio, HR = 1.57; 95% CI: 1.08—2.30; *p* = 0.020, Fig. 3) was shown.

**Fig. 4:**
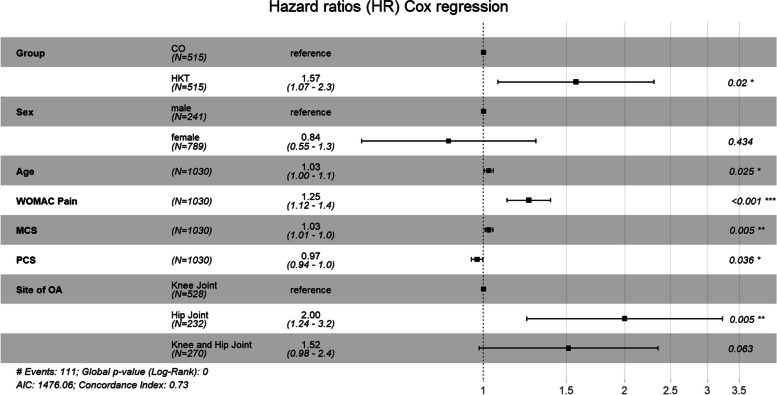
Hazard ratios cox regression model: risk factors for artificial joint replacement

In this model, a statistically significant increased risk for AJR was also associated with worse baseline pain and PCS, better baseline MCS, higher age, male sex and hip OA vs. knee OA (Fig. 4).

### Exercise-related outcomes (t3, HKT only)

#### Perceived benefit from the intervention/satisfaction with exercise instructors

289 (56%) participants of HKT rated the perceived benefit of the intervention with *high* or *very high*, 18 (3%) stated to have perceived *no* or *little benefit, and* 144 (28%) did not respond. The best categories for trainer competence (*very competent*), trainer motivation (*very engaged and motivated*) and recommendation of HKT to others (*definitely yes*) were selected by more than two third of all responders (Table 6).

**Table 6:** Perceived benefit from Hip and Knee Training (HKT) and satisfaction with exercise instructors, *n* (%)

	**Levels**					**Total** (***n*** (%))	**Missings** (***n*** (%) of ***n*** = 515)		
	**1**	**2**	**3**	**4**	**5**		**m. v. item**	**m. v. t3**	**thereof DO t3**
Overall perceived benefit (1–5) ^a^	111 (21.6)	178 (34.6)	64 (12.4)	16 (3.1)	2 (0.4)	371 (72.0)	4 (0.8)	140 (27.2)	64 (12.4)
Recommendation of HKT to others (1–4)	257 (49.9)	104 (20.2)	10 (1.9)	0 (0.0)	-	371 (72.0)	4 (0.8)	140 (27.2)	64 (12.4)
Competence of instructors (1–4)	285 (55.3)	81 (15.7)	6 (1.2)	0 (0.0)	-	372 (72.2)	3 (0.6)	140 (27.2)	64 (12.4)
Motivation of instructors (1–4)	303 (58.8)	67 (13.0)	1 (0.2)	0 (0.0)	-	371 (72.0)	4 (0.8)	140 (27.2)	64 (12.4)

^a^ Lower score indicate positive response. *m*. *v*. *item* Missing values for specific item, *m*. *v*. *t3,* Missing values for t3, *thereof* *DO t3,* (DO = Dropout), data lost to follow-up from t3 to t24)

#### Exercise adherence and reasons for non-attendance

Exercise adherence between t0 and t3 was reported by 72% of all HKT participants (*n* = 369). Thereof, 157 (43%) and 276 (75%) participants attended all scheduled group and home training sessions, respectively. One or more sessions were missed by 196 (53%, group) and 80 (22%, home) participants. The most frequently reported reasons for skipping training sessions were “family/work duties” (149 entries) and “experiencing pain” (72 entries). More details are given in Additional Information S12.

#### Exercise-related adverse events

Adverse events were common with *n* = 190 (37%) participants experiencing exercise-related pain because of HKT. For more information on pain frequency, duration and intensity refer to Additional Table S 13. No serious adverse events were reported to the principal investigators.

#### Intervention delivery

Up to the end of 2016, 88 health care professionals of the AOK-BW had been trained to instruct HKT groups. They were strongly encouraged to lead the exercise sessions according to the instructor manual and the exercise book. However, treatment fidelity was not monitored throughout the study period.

### Concomitant care (t3—t24)

In summary, 33–36% of the participants of CO explicitly stated to do a specific hip or knee exercise program offered by the AOK-BW or other providers (t0-t24, response rates 68–77%). For HKT, 23–41% of the subjects explicitly stated to attend a hip/knee-specific exercise program after the study intervention phase (t3-t24, response rates 44–60%). Additional lifestyle interventions of the AOK-BW (i. e. mind–body exercises, stretching, back strengthening exercises, nutrition and healthy weight) were utilized by 8–9% (CO) and 17–24% (HKT) during 24 months follow-up with response rates between 61–80%. More information on exercise- and lifestyle-related concomitant care is outlined in Additional Tables S 14 and S 15).

## Discussion

This trial demonstrates a statistically significant short-, mid- and long-term effectiveness of a land-based hip and knee training program (HKT). It was offered at more than 70 sites in the federal state of Baden-Wuerttemberg for customers of a health insurance company suffering from hip and/or knee OA. Pain was reduced in HKT in comparison to CO across all time-points with the largest differences in the short-term (estimated treatment differences (ETD) = 0.47 to 0.34; ES ≤ 0.22). Function did not improve in HKT, but worsening was less in comparison to control (CO) with ETDs = 0.22 to 0.33 and ES < 0.2 Meta-analyses on exercise therapy in OA show, that effect sizes for function are smaller than those reported for pain [5, 28, 29]. However, we do not have an explanation for the short-term decrease of physical function in the control group as OA progresses slowly [30].

Except for short-term effects on pain, treatment differences were below reported margins for the minimally clinically important difference between groups [31]. Looking at within-group differences for HKT, only pain at t3 and t12 was statistically significant from zero and mean values were much smaller than reported values for minimum clinical important differences [32]. Our treatment effects were also smaller compared to the latest Cochrane reviews on exercise therapy in OA reporting short-term improvements for pain and function [5, 6], and effects reported in a previous RCT evaluating the efficacy of the exercise intervention which was the blueprint of the HKT intervention under study [14].

These differences may be caused by the so-called efficacy-effectiveness gap which is attributed to the fact that most RCTs are optimized to determine efficacy and could therefore overestimate benefits [7]. Our trial was conducted in real-life without randomization and restrictions to standard care. Exercise-related standard care could have confounded superiority of HKT versus CO. We therefore conducted a sub-analysis to investigate whether treatment differences between HKT and participants of CO doing hip/knee-specific exercise (CO-exercise) would be even smaller, which was not the case. CO-exercise was more active in a health-oriented manner than HKT at baseline. Therefore, it cannot be ruled out that this patient group already benefited from exercise in the past and therefore did not show any additional treatment effects during the intervention period of our study. Another possible reason for the small treatment effects may be related to a potential lack of compliance towards the intended implementation of the exercise program. Treatment fidelity was not monitored in this trial and remains an open question. Lack of adherence can cause exercise effectiveness, as well. Adherence was assessed at t3 with a retrospective time window of three months. More than 25% of participants of HKT did not respond at all. The majority of responders missed one or more group sessions. Reports on adherence to home sessions was much higher, however a social desirability might have affected response behavior. These numbers indicate that the rather small treatment effects may also be due to insufficient exercise adherence.

Besides possible reasons for the small effects due to study-related reasons, recent data of a comprehensive individual patient data analysis on 31 RCTs including *n* = 4241 participants with hip and/or knee OA relativize previous findings and question clinical importance of treatment effects especially in the medium and long term with differences between exercise and non-exercise control of -3.77 points (95% CI: -5.97 to -1.57) and -3.43 points (95% CI: -5.18 to -1.69) for pain and -2.71 points (95% CI: -4.63 to -0.78) and -3.39 points (95% CI: -4.97 to -1.81) for function (scale 0 – 100 best to worst) [29]. These differences are similar to the study results of our primary analysis. Comparable results to our study were also reported in a pragmatic multi-center RCT in a primary care setting including 203 participants with hip OA who were recruited by general practitioners (GP) and randomized into an intervention group providing GP care, an information brochure and exercise (IG) or GP care and the brochure only (CO) [33]. The authors reported superiority of IG at three months with -3.7 points (95% CI: -7.3 to -0.2, ES = -0.23) for pain and -5.3 (95% CI: -8.9 to -1.6, ES = -0.31) for physical function (scale 0 – 100 best to worst). No statistically significant differences were found for 12-months follow-up (pain: *p* = 0.49, ES = -0.10; function: *p* = 0.25, ES = -0.17).

In contrast to the aforementioned results with questionable clinical importance, several nation-wide implemented community-based interventions for patients with knee or hip OA such as *BOA* (Sweden) [10], *AktivA* (Norway) [11], *GLA.D®* (Denmark) [12, 34] and *ESCAPE-pain* (United Kingdom) [13] provide evidence for sustainable pain reduction after having participated in the programs. The reported effects outreach those of our study with pain being reduced between 0.52 to 1.24 points after ceasing the intervention and 0.82 and 1.37 points after 12 months (reported numbers were transformed to a scale from 0–10 for a better comparison). All of these programs have in common that they scaled up evidence-based interventions combining supervised exercise instructions with patient education and that they used a national registry to register outcome data. As such, only complete case (CC) or all available data (AA) could be used for their evaluation. Although the effect sizes reported in our sensitivity analysis on CC and AA are larger compared to our primary analyses, effect sizes for pain reduction are still smaller in comparison to the other programs. Average baseline pain levels of patients participating in the national programs mentioned above were about 5.0 (scale 0–10 best to worst, transferred if necessary). In contrast, the average pain level of our intervention group was 3.1. Patients with only mild symptoms need less improvement to perceive a personal benefit as clinically important [35], and self-reports of participants of HKT showed, that more than half of them (highly) benefited from the intervention. Both facts underline a potential positive treatment effect from an individual perspective despite small mean changes from baseline for both primary outcomes.

We did not find a significant intervention effect on mental health or self-efficacy. Results are not surprising for mental health as MCS baseline values were comparable to the norm population. Regarding self-efficacy, previous studies have shown that participants of an exercise and education intervention increased self-confidence in their ability to cope with the consequences of arthritis [11, 36]. However, we used a generic scale not related to OA symptoms [22], thus a final statement on the effectiveness of the intervention towards the mastering of OA related complaints remains unknown. We were also interested in treatment effects on the health-oriented activity status. Despite similar baseline values for both groups, the amount of being active in a health-oriented manner (i. e. engaging in fitness activities or walking) decreased in participants of CO whereas mean values for HKT participants showed a slight increase after having completed the intervention. Although using different measures to assess health-oriented physical activity behavior, our results point in the same direction as a recent systematic review reporting small increases in physical activity for people with knee OA participating in exercise therapy in comparison to a control group in the short-term [37]. Our study also compared the number of first incidence of AJR during 24 months follow-up. Data were derived from the insurance database, thus being available even for participants lost to follow-up. Incidence for AJR was higher for HKT (13%) in comparison to CO (8.7%) during 24-months follow-up. Further risk factors for AJR were more pain, worse physical functioning, higher age, and having hip OA, which have been reported to be associated with higher surgery rates previously [38, 39]. However, higher risk for AJR after participating in an exercise intervention was not to be expected. Only few trials gave numbers on surgery rates after exercise interventions versus control. One study reported a statistically significant lower risk for joint replacement during a six-year follow-up period in 109 patients with hip OA who had participated in a structured exercise program before [40]. The percentage of participants undergoing joint replacement during 6-years follow-up was 40% and 57% for exercise and control, respectively. In another study investigating the effects of a four weeks manual therapy and exercise intervention versus subtherapeutic ultrasound in 83 patients with knee OA, 5% of patients in the treatment group and 20% of patients in the placebo group had undergone knee arthroplasty at 1 year (*p* = 0.039) [41]. However, 5-years follow-up data of a study comparing an intervention including patient education, supervised exercise and other non-surgical treatments with written advice in 100 patients with knee OA not being eligible for AJR at baseline revealed similar surgery rates of 30% and 36% for both treatments, respectively [42]. Data without a control group from the *BOA* and *GLA:D®* registries reported surgery rates of 30% for hip OA and 16% for knee OA during follow-up of two years after having participated in an exercise intervention [11, 39]. All these numbers show that AJR is a common treatment for many patients suffering from OA. Failure of conservative treatment options, the individual level of suffering of the patient and his personal wishes makes a significant contribution to the decision-making process whether surgery is indicated [43]. One may speculate that participants of HKT took the initiative to participate in the exercise program with the idea of counteracting OA symptoms, and if this treatment option failed, the next step to AJR could have been realized. This argument is underlined by the fact that mental health at baseline was higher in patients undergoing surgery, indicating that better mental well-being rather represents a driver for action than a hindrance. To obtain a more comprehensive view of the association between exercise and the decision to opt for surgery, more data are needed that do not only report on the incidence of AJR but also on individual and context factors that are related to this issue.

Structured land-based exercise deems appropriate for use by the majority of patients and safe for use in conjunction with other first-line and second-line treatments [1]. Still, a 1.8-fold relative risk of non-serious adverse events of exercises in patients with musculoskeletal complaints has been reported previously, but no increased risk of serious adverse events [44]. This statement can be confirmed with our data. Exercise-related pain was a side effect reported by one third of participants of HKT. Yet, only few participants indicated complaints lasting for longer than the next day and no serious adverse event was reported to the study team. However, pain is a commonly cited barrier to exercise [45] and was also the second most frequent reason for skipping an exercise session in our study. In addition, participants with higher pain levels or those suffering from multi-joint OA were also prone to prematurely drop out of the study. Best practice therapeutic exercise delivery involves adapting exercises according to the individual symptom state and providing information on strategies for managing short-term increases in pain during and after exercise [45, 46]. The exercise manual for HKT comprised information on how to cope with exercise-induced pain by adapting dosage parameters or choosing another exercise with a similar aim. However, we have no information to what extent the intervention was delivered as planned. It is therefore important to gain more knowledge on the feasibility and effectiveness of individualized exercise modifications in scaled-up interventions and their potential to improve adherence.

### Strengths and limitations

An important strength of the study is the inclusion of a control group in a community-based setting. Although groups are not randomized, propensity score matching allowed us to match the participants of the intervention according to relevant characteristics, including but not limited to age, sex, site of OA, and health care costs. However, this strength of a matched control group may also be a source of potential selection bias, as recruitment strategies differ remarkably between groups. Participants of HKT decided to participate in a strengthening program with the aim to counteract OA symptoms. We controlled for many OA related confounders, however psychological aspects such as the state of change for behavioral interventions, treatment expectations or OA related self-efficacy and other potential confounders were not controlled for. This might have influenced the results of our study.

The imputation of missing data is another strength of the study. It is rarely applied in pragmatic studies in a real-world context, however it enabled us to handle the increasing number of missings throughout the complete study period. As such, results are not only based on all available data that are prone to those responding better to treatment as demonstrated in our sensitivity analysis. Despite the advantage of imputing missing values, we acknowledge that we did not perform complex analyses under a missing not at random (MNAR) mechanism to address potential violations of the assumed missing at random (MAR) assumption for our MI procedure. A further limitation of this trial is the percentage of missings for the primary endpoint at t3 with 30% for HKT and 21% for CO. However, only 12% (HKT) and 7% (CO) of them were drop-outs not responding to a later time point. We do not have an explanation for this finding at t3 as data assessments were automatized by sending out postal questionnaires at pre-defined dates according to the baseline assessment.

Lastly, we have to acknowledge that a relevant number of participants of CO reported to be engaged in hip/knee-specific exercises. We conducted a subgroup analysis to investigate the impact of this finding on estimated mean differences between groups. Results did not change and we can therefore conclude that concomitant care of exercising in the control group did not confound our study results.

## Conclusions

This trial was conducted in a real-life setting to evaluate short-, mid- and long-term effectiveness of an exercise intervention specifically designed for patients with hip or knee OA in comparison to a control. Due to the trial design, a high external validity and thus generalizability of study results can be assumed. The hip and knee training group was superior to the control in terms of pain reduction and better physical functioning. However, treatment differences were smaller than those reported in previous trials, and—except for short-term effects on pain—below reported margins for the minimally clinically important difference [32]. Despite these findings, the majority of participants of the intervention rated the perceived benefit of the intervention with *high* or *very high.* Therefore, the next step is to conduct a responder analysis and to explore personal contextual factors that differentiate responders from non-responders to allow a better understanding of relevant prerequisites for successful exercise participation [17]. We also recorded a relevant number of patients prematurely dropping out of the study as well as a higher proportion of participants of the intervention group opting for joint surgery during follow-up. It is therefore important to gain more knowledge on reasons for prematurely ceasing the health care offer as to find ways to make the intervention feasible for the majority of patients with hip or knee OA. Future quasi-experimental trials should further provide information on long-term treatment effects in real-life scenarios that also include numbers and reasons for joint replacement to investigate whether exercise advances the decision for joint surgery in some and postpones it in others.

### Supplementary Information

**Additional File 1:** **Additional Information S1.** Deviations from the study protocol. **Additional Table S2.** In-and exclusion criteria for participation in the study. **Additional Table S3.** Description of home exercises for patients with knee OA. **Additional Table S4.** Description of home exercises for patients with hip OA. **Additional Table S5.** Baseline characteristics before and after propensity score final matching of the hip and knee training group (HKT) and control (CO). **Additional Table S6.** Baseline characteristics of completers vs. dropouts. **Additional Table S7.** Socio-demographic characteristics at baseline of the matched pairs study population (*n*=1030). **Additional Table S8.** Primary and sensitivity analyses with fixed-effect ANOVA tables of the linear mixed models for WOMAC pain and function (t0 - t3). **Additional Table S9.** Primary and sensitivity with fixed-effect ANOVA tables of the linear mixed models for WOMAC pain and function (t0 - t24). **Additional Table S10.** Sensitivity analysis for WOMAC pain and function at t3, t6, t12 and t24 months change from baseline (cfb, 95% CI). **Additional Table S11.** Comparison of estimated treatment difference CO – HKT for primary and secondary analysis for WOMAC pain and function at t3, t6, t12 and t24 months change from baseline (cfb, 95% CI). **Additional Information S12.** Exercise adherence and reasons for non-attendance. **Additional Table S13.** Frequency of self-reported exercise-related pain during HKT (t3), n (%). **Additional Table S14.** Concomitant care related to hip and knee training during the previous follow-up period, n (% of *n* = 515/group). **Additional Table S15.** Concomitant care related to other health care offers of the AOK-BW and else during the previous follow-up period, n (% of *n* = 515/group). **Additional Table S16.** Baseline characteristics of complete case population (t0, t3) of HKT and subgroup CO-exercise (CO participants having reported to engage in hip/knee joint-specific exercises between t0 and t3). **Additional Table S17.** Within-group estimates of change from baseline (cfb, 95% CI) and the according between-group estimated treatment differences (ETDs) at t3 for WOMAC pain and function of HKT and subgroup CO-exercise (CO participants having reported to engage in hip/knee joint-specific exercises between t0 and t3).

**Additional File 2.** CERT (Consensus on Exercise Reporting Template) checklist: A Checklist for what to include when reporting exercise programs.

**Additional File 3.** 2017 CONSORT checklist of information to include when reporting a randomized trial assessing nonpharmacologic treatments (NPTs)*. Modifications of the extension appear in italics and blue.

## Data Availability

The datasets supporting the conclusions of this article are available from the corresponding author on reasonable request.

## Abbreviations

AA: All available data
AJR: Artificial joint replacement
AktivA: Active with OsteoArthritis
AOK-BW: Allgemeine Ortskrankenkasse Baden-Wuerttemberg
BMI: Body mass index
BOA: Better life with Osteoarthritis
CC: Complete case
CERT: Consensus on exercise reporting template
Cfb: Change from baseline
CI: Confidence intervals
CO: Control
ESCAPE-pain: Evidence-based complex intervention for knee and hip osteoarthritis
ETD: Estimated treatment difference
EMM: Estimated marginal means
ES: Effect size
FU: Follow-up
GLA:D®: Good life with Osteoarthritis in Denmark
GP: General practictioner
GSE: General self-efficacy scale
HKT: Hip and knee training
Ho-AS: Health-oriented activity status
HR: Hazard ratio
ICD: International classification of diseases
IG: Information brochure and exercise
LMM: Linear mixed model
MCS: Mental component score
MAR: Missing at random
MI: Multiple imputation
MVC: Maximum voluntary contraction
NRS: Numerical rating scale
OA: Osteoarthritis
PCS: Physical component score
PH: Proportional hazard
PSM: Propensity score matching
RCT: Randomized controlled trials
REML: Restricted maximum likelihood estimation
SC: Standard care
SE: Standard Error
SMD: Standardized mean difference
VR-12: Veterans RAND 12-item health survey
WOMAC®: Western Ontario and McMaster universities osteoarthritis index
