# Supplementary material for: Effectiveness of an 11-week exercise intervention for patients with hip or knee osteoarthritis: results of a quasi-experimental pragmatic trial
Source: BMC Sports Sci Med Rehabil. 2024 Jan 20;16:24. doi: 10.1186/s13102-023-00779-0 (PMC10799538; doi:10.1186/s13102-023-00779-0)
Supplement: Supplementary file 1 — Additional File 1: Additional Information S1. Deviations from the study protocol. Additional Table S2. In-and exclusion criteria for participation in the study. Additional Table S3. Description of home exercises for patients with knee OA. Additional Table S4. Description of home exercises for patients with hip OA. Additional Table S5. Baseline characteristics before and after propensity score final matching of the hip and knee training group (HKT) and control (CO). Additional Table S6. Baseline characteristics of completers vs. dropouts. Additional Table S7. Socio-demographic characteristics at baseline of the matched pairs study population (n=1030). Additional Table S8. Primary and sensitivity analyses with fixed-effect ANOVA tables of the linear mixed models for WOMAC pain and function (t0 - t3). Additional Table S9. Primary and sensitivity with fixed-effect ANOVA tables of the linear mixed models for WOMAC pain and function (t0 - t24). Additional Table S10. Sensitivity analysis for WOMAC pain and function at t3, t6, t12 and t24 months change from baseline (cfb, 95% CI). Additional Table S11. Comparison of estimated treatment difference CO – HKT for primary and secondary analysis for WOMAC pain and function at t3, t6, t12 and t24 months change from baseline (cfb, 95% CI). Additional Information S12. Exercise adherence and reasons for non-attendance. Additional Table S13. Frequency of self-reported exercise-related pain during HKT (t3), n (%). Additional Table S14. Concomitant care related to hip and knee training during the previous follow-up period, n (% of n = 515/group). Additional Table S15. Concomitant care related to other health care offers of the AOK-BW and else during the previous follow-up period, n (% of n = 515/group). Additional Table S16. Baseline characteristics of complete case population (t0, t3) of HKT and subgroup CO-exercise (CO participants having reported to engage in hip/knee joint-specific exercises between t0 and t3). Additional Table S17. [file 13102_2023_779_MOESM1_ESM.docx]

**Additional Files**

**Additional Information S1:** Deviations from the study protocol.

**Additional Table S2**: In-and exclusion criteria for participation in the study.

**Additional Table S3:** Description of home exercises for patients with knee OA.

**Additional Table S4:** Description of home exercises for patients with hip OA.

**Additional Table S5**: Baseline characteristics before and after propensity score final matching of the hip and knee training group (HKT) and control (CO).

**Additional Table S6**: Baseline characteristics of completers vs. dropouts.

**Additional Table S7**: Socio-demographic characteristics at baseline of the matched pairs study population (n=1030).

**Additional Table S8**: Primary and sensitivity analyses with fixed-effect ANOVA tables of the linear mixed models for WOMAC pain and function (t0 - t3).

**Additional Table S9**: Primary and sensitivity analyses with fixed-effect ANOVA tables of the linear mixed models for WOMAC pain and function (t0 - t24).

**Additional Table S10:** Sensitivity analyses for WOMAC pain and function at t3, t6, t12 and t24 months change from baseline (cfb, 95% CI).

**Additional Table S11:** Comparison of estimated treatment difference CO – HKT for primary and sensitivity analyses for WOMAC pain and function at t3, t6, t12 and t24 months change from baseline (cfb, 95% CI).

**Additional Information S12:** Exercise adherence and reasons for non-attendance.

**Additional Table S13:** Frequency of self-reported exercise-related pain during HKT (t3), n (%).

**Additional Table S14:** Concomitant care related to hip and knee training during the previous follow-up period, n (% of n = 515/group).

**Additional Table S15:** Concomitant care related to other health care offers of the AOK-BW and else during the previous follow-up period, n (% of n = 515/group).

**Additional Table S16:** Baseline characteristics of complete case population (t0, t3) of HKT and subgroup CO-exercise (CO participants having reported to engage in hip/knee joint-specific exercises between t0 and t3).

**Additional Table S17:** Within-group estimates of change from baseline (cfb, 95% CI) and the according between-group estimated treatment differences (ETDs) at t3 for WOMAC pain and function of HKT and subgroup CO-exercise (CO participants having reported to engage in hip/knee joint-specific exercises between t0 and t3).

**Additional Information S1:** Deviations from the study protocol^1^.

**METHODS**

*Recruitment and participant flow*

Recruitment of the participants was intended within one year, yet had to be extended due to delay. Nevertheless, the estimated sample size could not be reached.

*Sample Size*

The sample size was originally estimated considering cluster effects of subjects within treatment groups (n = 4 each group, ICC = 0.33, variance inflation factor 2 for the intervention). However, we did not cluster by treatment groups because information on exercise groups were not available.

*Blinding*

Statistician blinding could not be warranted due to the necessary preparation of the baseline data of the intervention group for the propensity score matching.

*Interventions*

There is no information about whether all participants of the intervention group were requested to refrain from seeking other forms of treatment during the eleven week-intervention period from t0 to t3. However physical activities and the utilization of other health care offers of the AOK-BW along the study period was monitored via self-administered questionnaires for participants of HKT and CO.

*Primary Outcomes*

Changes had to made to the primary trial outcome after the trial commenced as the intended procedure was not feasible for analytical reasons: According to the study protocol participants with limited pain and functional impairment (both WOMAC subscales < 1.5 points) at study entry were to be excluded in the primary analyses (t0, t3), however, a 1:1 matched-pair design with equal group sizes in HKT and CO group could not be guaranteed applying this criterium. We therefore included all eligible participants regardless of their baseline scores.

*Secondary Outcomes*

*General self-efficacy*: In difference to the study protocol, general self-efficacy was not expressed as a sum score of all items (score range 10-40), but a mean score of all items. A mean score was calculated when at least six items were present allowing for a minority of missing values within the scale.

*Physical activity status:* We did not include this measure into analysis as it is quite similar to the outcome measure *health-oriented activity status*.

*Statistical analyses*

*Primary analysis* According to the above-mentioned change to the primary outcomes, the primary analysis has changed as well and refers to the complete sample regardless of baseline pain and functional impairment.

*Sensitivity Analyses:* We refrained from carrying out a sensitivity analysis using last observation carried forward (LOCF), as this technique is considered conservative and outdated ^2^. Instead, an analysis on all available data was used in addition to the pre-specified complete case analysis.

*Non-considered analysis:* Responder analysis according to the OMERACT-OARSI set of responder criteria as well as exploratory analysis of prognostic factors for response to treatment as outlined in the study protocol are not part of this publication.

*Exploratory* *analysis* of risk ratio for artificial joint replacement between Intervention and Control as well as the analysis of risk factors for joint replacement were not pre-specified in the study protocol. In addition to the explanations as outlined in the section *Methods – Statistical Analysis*, the following procedure was used to reject potential co-variates for the risk of joint replacement from the final model, taking into consideration not to include redundant information and covariates with many missing to avoid list-wise deletion with the consequence of reducing sample size for this analysis:

- Correlation between the *General Self-efficacy scale (GSE)* and the *Mental Component Scale (MCS)* at baseline was r = .53, and *GSE* was missing for n = 26. ⇒ *GSE* was disregarded from further analysis and not included in the final model.
- Correlation between the *Physical Component Scale (PCS)* and *WOMAC Function* at baseline was r = .70, and between *WOMAC Function* and *WOMAC Pain* at baseline = .85. Pain seems extremely relevant for AJR and *WOMAC function* was missing for n = 20.

⇒ *WOMAC function* was disregarded from further analysis and not included in the final model.

- A preliminary analysis revealed insignificance for *Health-oriented activity status (Ho-AS)*, *BMI* and *AJR at baseline in any hip/knee joint*. Ho-AS and BMI were missing for n = 19 and n = 37, respectively.

⇒ *(Ho-AS)*, *BMI* and *AJR at baseline* were disregarded from further analysis and not included in the final model.

*Exploratory analysis* of the comparison of WOMAC pain and WOMAC function at t3 versus baseline for complete cases of HKT versus subsample CO-exercise. CO-exercise was defined as participants of CO having reported to engage in any hip/knee-specific exercise between t0 and t3. This exploratory analysis was asked for by a reviewer of the manuscript.

*Interims Analysis:* We conducted an interims analysis comparing the intervention group of this trial^1^ with the intervention group of another trial^3^ without including the control group of both trials at t3 for a report towards the health insurance company and after t24 because of a delay of the collection of control group data^4^. This step occurred prior to propensity score matching.

**Other**

*Expert report:* The study was not assessed by a third party as outlined in the study protocol because it does no longer correspond to common practice of the AOK-BW and is not common practice for clinical studies either.

**References of Additional File S1**

1. Krauss I, Mueller G, Haupt G, Steinhilber S, Janssen P, Jentner N, et al. Efficacy and efficiency of an 11-week exercise intervention for patients with hip or knee osteoarthritis: A protocol for a controlled study in the context of health service research. BMC Public Health 2016; 16: 367.

2. Israni A. Advances in Multilevel Modeling for Educational Research: Addressing Practical Issues Found in Real-World Applications. 2014.

3. Krauss I, Muller G, Steinhilber B, Haupt G, Janssen P, Martus P. Effectiveness and efficiency of different weight machine-based strength training programs for patients with hip or knee osteoarthritis: a protocol for a quasi-experimental controlled study in the context of health services research. BMJ Open Sport Exerc Med 2017; 3: e000291.

4. Roesel I, Krauss I, Martus P, Steinhilber B, Mueller G. Comparison of a Group-/Home-Based and a Weight-Machine-Based Exercise Training for Patients with Hip or Knee Osteoarthritis-A Secondary Analysis of Two Trial Interventions in a Real-World Context. Int J Environ Res Public Health 2022; 19.

**Additional Table S2**: In-and exclusion criteria for participation in the study. Modified version of the original source: Krauss I, et al. BMJ Open Sport Exerc Med 2017; 3: e000291.

| **Inclusion criteria** |
| --- |
| - Hip or knee complaints (OA, state after joint-preserving or joint-replacing surgery and corresponding curative care, pre-arthritic conditions) - Physical and mental ability to participate in the interventional program and to answer self-administered questionnaires - Self-reported lifetime prevalence of hip and/or knee OA diagnosed by a medical practitioner |
| - Insurance holder of the insurance company offering the exercise program for two or more years |
| **Exclusion criteria** |
| - Significant established osteoporosis requiring treatment, previous spontaneous or low impact fracture |
| - Co-morbidities leading to major impairments in everyday life and representing contra-indications for physical activities |
| - Artificial joint replacement at the knee and/or hip joint within the last 6 months |
| - Artificial joint replacement at the knee and/or hip joint with instable anchoring |
| - Artificial joint replacement at the knee and/or hip joint with radiologic signs of implant loosening |
| - Current pain at rest or with activity due to artificial joint replacement at the knee and/or hip joint |
| - Luxation as an adverse event of artificial hip replacement |
| - Acute joint inflammation at the knee and/or hip joint |
| - Musculo-skeletal surgery at the lower extremity within the last 3 months |
| - Regular use of gait aids |
| - Self-reported acute illness |
| - Insufficient German language ability for self-administered study questionnaires and for the understanding of the verbal exercise instructions and written exercise descriptions and intervention materials. |
| - Current employment in the health care insurance |

**Additional Table S3:** Description of home exercises for patients with knee OA (exercises for postural control are not included in the table).

| **Type**^[[1]](#footnote-1)^ **/ No** | **Exercise** | **Position** | **M-Plane**^[[2]](#footnote-2)^ | **RoI**^[[3]](#footnote-3)^ | **Muscles** | **Tools**^[[4]](#footnote-4)^ |
| --- | --- | --- | --- | --- | --- | --- |
| **Week 1** | | | | | | |
| MM1 (K) | Mobilization | Supine | S | Knee/Ankle |  |  |
| MM2 (K) | Mobilization | Supine | S | Hip/Knee |  |  |
| MM3 (K) | Stretching | Supine | S | Ankle | Calf |  |
| MM4 (H) | Motor learning | Supine | C/T | Hip | Adductors | EB |
| MM5 (H) | Motor learning | Supine | S | Hip/Knee | Hip extensors  Knee flexors | EB |
| MM6 (K) | Mobilization | Seated | S | Hip/Knee |  |  |
| MM7 (H) | Motor learning | Seated | S | Hip | Core |  |
| MM8 (H) | Motor learning | Seated | S | Hip/Knee/Ankle | Hip flexors/Calf |  |
| MM9 (K) | Mobilization | Seated | T | Knee |  |  |
| MM10 (K) | Mobilization | Seated | S | Knee |  |  |
| **Week 2** | | | | | | |
| MM11 (H) | Motor learning | Seated | S | Contact area foot | Lower extremity |  |
| MM12 (H) | Motor learning | Seated | T | Contact area foot | Lower extremity |  |
| MM13 (H) | Stretching | Seated | S | Hip/Knee/Ankle | Hamstrings/Calf |  |
| MM14 (H) | Motor learning | Standing |  | Contact area foot | Lower extremity |  |
| MM15 (H) | Motor learning | Standing | F | Contact area foot | Lower extremity |  |
| MM16 (H) | Motor learning | Standing | F | Contact area foot | Lower extremity |  |
| MM17 (K) | Mobilization | Standing | S | Hip/Knee |  | Stick |
| MM18 (H) | Motor learning | Standing | S | Hip/Knee/Ankle | Core  Lower extremity |  |
| MM19 (H) | Stretching | Standing | S | Hip/Knee | Hip flexors  Knee extensors | RB |
| MM20 (H) | Stretching | Standing | S | Hip/Knee | Hamstrings/Calf |  |
| **Week 3** | | | | | | |
| MM21 (K) | Motor learning | Supine | S | Hip/Knee | Extensors | EB |
| MM22 (K) | Motor learning | Supine | S | Knee | Extensors |  |
| MM23 (H) | Motor learning | Supine | S | Hip/Knee | Hamstrings |  |
| MM24 (K) | Stretching | Supine | S | Hip/Knee | Hamstrings/Calf |  |
| MM25 (K) | Motor learning | Prone | S | Knee | Hamstrings |  |
| MM26 (K) | Motor learning | Prone | S | Hip/Knee | Lower extremity |  |
| MM27 (H) | Motor learning | Step position |  | Contact area foot | Lower extremity |  |
| MM28 (H) | Motor learning | Step position |  | Contact area foot | Lower extremity |  |
| MM29 (H) | Motor learning | Step position |  | Contact area foot | Lower extremity |  |
| MM30 (H) | Mobilization | Step position | S | Hip/Knee | Flexors |  |

**Additional Table S3** (continued)**.**

| **Type**^[[5]](#footnote-5)^ **/ No** | **Exercise** | **Position** | **M-Plane**^[[6]](#footnote-6)^ | **RoI**^[[7]](#footnote-7)^ | **Muscles** | **Tools**^[[8]](#footnote-8)^ |
| --- | --- | --- | --- | --- | --- | --- |
| **Week 4** | | | | | | |
| S1 (H) | Strengthening | Supine | S | Hip | Extensors |  |
| S2 (H) | Strengthening | Supine | S | Hip | Flexors |  |
| S3 (K) | Strengthening | Prone | S | Knee | Flexors |  |
| S4 (K) | Strengthening | Supine | S | Knee | Extensors | EB |
| **Week 5** | | | | | | |
| S5 (H) | Strengthening | Standing | S | Hip | Extensors |  |
| S6 (H) | Strengthening | Seated | S | Hip | Flexors |  |
| S7 (K) | Strengthening | Standing | S | Knee | Flexors | Sticks |
| S8 (K) | Strengthening | Seated | S | Knee | Extensors |  |
| **Week 6** | | | | | | |
| First home training session according to week 4, second home training session according to week 5. | | | | | | |
| **Week 7** | | | | | | |
| One of the exercises S1-S8 for each muscle group (hip extensors, hip flexors, knee extensors, knee flexors). Type of exercise can be chosen according to personal preferences - 4 exercises in total. | | | | | | |
| **Week 8-11: First home training session of the week** | | | | | | |
| S11 (K) | Strengthening | Prone | S | Knee | Flexors | WC |
| S12 (K) | Strengthening | Supine | S | Knee | Extensors | WC/EB |
| S13 (K) | Strengthening | Standing | S | Knee | Flexors | Sticks/WC |
| S14 (K) | Strengthening | Seated | S | Knee | Extensors | WC |
| **Week 8-11: Second home training session of the week** | | | | | | |
| S17 (H) | Strengthening | Standing | S | Hip | Extensors | EB |
| S18 (H) | Strengthening | Standing | S | Hip | Flexors | EB |
| S19 (K) | Strengthening | Standing | S | Knee | Extensors | Sticks |
| S20 (K) | Strengthening | Standing | S | Hip/Knee | Extensors | Sticks |

**Additional Table S4:** Description of home exercises for patients with hip OA (exercises for postural control are not included in the table).

| **Type**^[[9]](#footnote-9)^ **/ No** | **Exercise** | **Position** | **M-Plane**^[[10]](#footnote-10)^ | **RoI**^[[11]](#footnote-11)^ | **Muscles** | **Tools**^[[12]](#footnote-12)^ |
| --- | --- | --- | --- | --- | --- | --- |

| **Week 1** | | | | | | |
| --- | --- | --- | --- | --- | --- | --- |
| MM1 (H) | Mobilization | Supine | S | Hip/Lumbar spine |  |  |
| MM2 (H) | Mobilization | Supine | S | Hip |  |  |
| MM3 (H) | Mobilization | Supine | C/T | Hip |  |  |
| MM4 (H) | Motor learning | Supine | C/T | Hip | Adductors | EB |
| MM5 (H) | Motor learning | Supine | S | Hip/Knee | Hip extensors  Knee flexors | EB |
| MM6 (H) | Mobilization | Seated | S | Hip/Lumbar spine |  |  |
| MM7 (H) | Motor learning | Seated | S | Hip | Core |  |
| MM8 (H) | Motor learning | Seated | S | Hip/Knee/Ankle | Hip flexors/Calf |  |
| MM9 (H) | Mobilization | Seated | C/T | Hip |  |  |
| MM10 (H) | Mobilization | Seated | T | Hip/Lumbar spine |  |  |
| **Week 2** | | | | | | |
| MM11 (H) | Motor learning | Seated | S | Contact area foot | Lower extremity |  |
| MM12 (H) | Motor learning | Seated | T | Contact area foot | Lower extremity |  |
| MM13 (H) | Stretching | Seated | S | Hip/Knee/Ankle | Hamstrings/Calf |  |
| MM14 (H) | Motor learning | Standing |  | Contact area foot | Lower extremity |  |
| MM15 (H) | Motor learning | Standing | F | Contact area foot | Lower extremity |  |
| MM16 (H) | Motor learning | Standing | F | Contact area foot | Lower extremity |  |
| MM17 (H) | Stretching | Standing | F | Hip | Adductors |  |
| MM18 (H) | Motor learning | Standing | S | Hip/Knee/Ankle | Core  Lower extremity |  |
| MM19 (H) | Stretching | Standing | S | Hip/Knee | Hip flexors  Knee extensors | RB |
| MM20 (H) | Stretching | Standing | S | Hip/Knee | Hamstrings/Calf |  |
| **Week 3** | | | | | | |
| MM21 (H) | Mobilization | Supine | T | Hip |  |  |
| MM22 (H) | Mobilization | Supine | C | Hip/Lumbar spine |  |  |
| MM23 (H) | Motor learning | Supine | S | Hip/Knee | Hamstrings |  |
| MM24 (H) | Mobilization | On the side | S | Hip/Lumbar spine |  |  |
| MM25 (H) | Motor learning | On the side | T/C | Hip | External rotators  Abductors |  |
| MM26 (H) | Motor learning | On the side | T/C | Hip/Knee | External rotators  Abductors |  |
| MM27 (H) | Motor learning | Step position |  | Contact area foot | Lower extremity |  |
| MM28 (H) | Motor learning | Step position |  | Contact area foot | Lower extremity |  |
| MM29 (H) | Motor learning | Step position |  | Contact area foot | Lower extremity |  |
| MM30 (H) | Motor learning | Step position | S | Hip/Knee | Flexors |  |

**Additional Table S4** (continued)**.**

| **Type**^[[13]](#footnote-13)^ **/ No** | **Exercise** | **Position** | **M-Plane**^[[14]](#footnote-14)^ | **RoI**^[[15]](#footnote-15)^ | **Muscles** | **Tools**^[[16]](#footnote-16)^ |
| --- | --- | --- | --- | --- | --- | --- |
| **Week 4** | | | | | | |
| S1 (H) | Strengthening | Supine | S | Hip | Extensors |  |
| S2 (H) | Strengthening | Supine | S | Hip | Flexors |  |
| S3 (H) | Strengthening | Supine | S | Hip | Abductors | RB |
| S4 (H) | Strengthening | Supine | S | Hip | Adductors | EB |
| **Week 5** | | | | | | |
| S5 (H) | Strengthening | Standing | S | Hip | Extensors |  |
| S6 (H) | Strengthening | Seated | S | Hip | Flexors |  |
| S7 (H) | Strengthening | Seated | S | Hip | Abductors | RB |
| S8 (H) | Strengthening | Seated | S | Hip | Adductors | EB |
| **Week 6** | | | | | | |
| First home training session according to week 4, second home training session according to week 5. | | | | | | |
| **Week 7** | | | | | | |
| One of the exercises S1-S8 for each muscle group (hip extensors, hip flexors, hip abductors, hip adductors). Type of exercise can be chosen according to personal preferences - 4 exercises in total. | | | | | | |
| **Week 8-11: First home training session of the week** | | | | | | |
| S9 (H) or S13 (H) | Strengthening | Supine or Stand | S | Hip | Extensors | WC |
| S10 (H) or S14 (H) | Strengthening | Supine or Seated | S | Hip | Flexors | WC  WC |
| S11 (H) or S15 (H) | Strengthening | Supine or Seated | S | Hip | Abductors | RB  RB |
| S12 (H) or S16 (H) | Strengthening | Supine or Seated | S | Hip | Adductors | EB  EB |
| **Week 8-11: Second home training session of the week** | | | | | | |
| S17 (H) | Strengthening | Standing | S | Hip | Extensors | EB |
| S18 (H) | Strengthening | Standing | S | Hip | Flexors | EB |
| S19 (H) | Strengthening | Standing | S | Hip | Abductors | EB |
| S20 (H) | Strengthening | Standing | S | Hip | Adductors | EB |

**Additional Table S5**: Baseline characteristics before and after propensity score final matching of the hip and knee training group (HKT) and control (CO).

|  | before matching | | | | | |  | after matching | | | | | |
| --- | --- | --- | --- | --- | --- | --- | --- | --- | --- | --- | --- | --- | --- |
|  | HKT | | CO | |  |  |  | HKT | | CO | |  |  |
|  | Mean | SD | Mean | SD | SMD | *p* |  | Mean | SD | Mean | SD | SMD | *p* |
| Propensity Score | 0.21 | 0.09 | 0.16 | 0.08 | 0.616 | <.001 |  | 0.20 | 0.08 | 0.20 | 0.08 | 0.015 | 0.810 |
| *Age* | 63.50 | 9.56 | 63.50 | 9.13 | 0.000 | 0.999 |  | 63.55 | 9.49 | 63.73 | 9.01 | -0.020 | 0.752 |
| *Sex (1= Female)* | 0.77 | 0.42 | 0.72 | 0.45 | 0.112 | 0.015 |  | 0.76 | 0.43 | 0.77 | 0.42 | -0.014 | 0.825 |
| BMI | 27.84 | 4.56 | 28.43 | 5.06 | -0.119 | 0.008 |  | 27.89 | 4.55 | 27.74 | 4.92 | 0.031 | 0.615 |
| Womac pain | 3.16 | 1.99 | 2.50 | 2.19 | 0.305 | <.001 |  | 3.14 | 1.99 | 3.16 | 2.31 | -0.009 | 0.883 |
| Womac function | 2.79 | 1.93 | 2.18 | 2.01 | 0.307 | <.001 |  | 2.78 | 1.93 | 2.74 | 2.20 | 0.019 | 0.766 |
| Quality-Adjusted Life Years (QALY) | 0.66 | 0.10 | 0.70 | 0.10 | -0.315 | <.001 |  | 0.66 | 0.09 | 0.66 | 0.10 | -0.004 | 0.945 |
| Participation in health activity programs (5= highest) | 2.90 | 0.87 | 3.10 | 1.02 | -0.204 | <.001 |  | 2.91 | 0.87 | 2.90 | 0.95 | 0.013 | 0.837 |
| General Self-Efficacy Scale | 3.07 | 0.56 | 3.16 | 0.54 | -0.167 | <.001 |  | 3.08 | 0.55 | 3.07 | 0.56 | 0.009 | 0.879 |
| *Quantity of Co-Morbidity unspecific tm24-t0** | 0.59 | 1.30 | 0.52 | 1.07 | 0.064 | 0.238 |  | 0.60 | 1.31 | 0.51 | 1.07 | 0.073 | 0.240 |
| *Morbidity Group Knee Hip tm24-t0 (1= yes)* | 0.01 | 0.12 | 0.02 | 0.14 | -0.049 | 0.245 |  | 0.01 | 0.12 | 0.01 | 0.08 | 0.079 | 0.204 |
| PCS90** | 38.16 | 8.32 | 41.95 | 8.90 | -0.430 | <.001 |  | 38.36 | 8.15 | 38.62 | 8.74 | -0.032 | 0.611 |
| MCS90** | 49.97 | 11.26 | 50.36 | 10.76 | -0.036 | 0.455 |  | 50.00 | 11.19 | 49.70 | 10.88 | 0.027 | 0.660 |
| Affected Joint**^e^ (1= hip, 2=knee, 3= both) | 1.75 | 0.84 | 1.67 | 0.81 | 0.107 | 0.030 |  | 1.75 | 0.84 | 1.75 | 0.84 | 0.000 | 1.000 |
| *Artificial joint replacement^e^ (1= yes)* | 0.13 | 0.33 | 0.25 | 0.43 | -0.294 | <.001 |  | 0.13 | 0.33 | 0.13 | 0.33 | 0.000 | 1.000 |
| Unspecific costs tm12-t0 | 2793 | 3601 | 2614 | 4084 | 0.045 | 0.349 |  | 2786 | 3606 | 2734 | 4408 | 0.013 | 0.837 |
| *Unspecific costs tm24-t0* | 5496 | 6012 | 5493 | 7309 | 0.000 | 0.992 |  | 5504 | 6038 | 5541 | 6973 | -0.006 | 0.929 |
| Specific costs tm12-t0 | 770 | 1854 | 713 | 1991 | 0.029 | 0.547 |  | 758 | 1828 | 623 | 1714 | 0.076 | 0.225 |
| *Specific costs tm24-t0* | 1330 | 2556 | 1695 | 3200 | -0.118 | 0.004 |  | 1322 | 2546 | 1129 | 2206 | 0.081 | 0.193 |
| Unspecific days of disability tm12-t0 | 11.6 | 39.6 | 11.6 | 34.8 | 0.001 | 0.987 |  | 11.4 | 39.4 | 10.2 | 29.7 | 0.033 | 0.592 |
| *Unspecific days of disability tm24-t0* | 21.2 | 61.2 | 23.6 | 57.7 | -0.042 | 0.380 |  | 21.0 | 61.2 | 20.5 | 52.0 | 0.009 | 0.888 |
| Specific days of disability tm12-t0 | 2.5 | 14.2 | 2.2 | 16.7 | 0.014 | 0.772 |  | 2.2 | 12.6 | 2.7 | 18.7 | -0.034 | 0.589 |
| *Specific days of disability tm24-t0* | 4.1 | 23.2 | 5.7 | 30.6 | -0.053 | 0.187 |  | 3.9 | 22.4 | 4.7 | 30.1 | -0.031 | 0.618 |

Note. Caliper: 0,22, costs in EUR, deviations and additions to the protocol: *not included in psm, because costs data already depict comorbidity, also not included: Habitual physical and sports activity, because well balanced (smd= 0,031, p= 0,34) without psm,** additionally included in psm, because they depict general health status (PCS90, MCS90), important for the comparability of the groups (affected joint), ^e^ exact matching, italics = variables that have been included also in the first matching (recruitment of the control group).

**Additional Table S6**: Baseline characteristics of completers vs. dropouts.

| **Variable** | **Overall (n = 1030)** | | | | | | |  | **By intervention group** | | | | | |  |
| --- | --- | --- | --- | --- | --- | --- | --- | --- | --- | --- | --- | --- | --- | --- | --- |
|  |  |  | |  | |  | |  | **HKT (n = 515)** | | |  | **CO (n = 515)** | |  |
|  | **Overall**  n = 1030 | | **Completers**  n = 691  (67.1%) | | **Dropouts**  n = 339  (32.9%) | | **p** |  | **Completers**  n = 312  (60.6%) | **Dropouts**  n = 203  (39.4%) | **p** |  | **Completers**  n = 379  (73.6%) | **Dropouts**  n = 136  (26.4%) | **p** |
| **Sex, n (%)*** |  | |  | |  | |  |  |  |  |  |  |  |  |  |
| Male | 241 (23.4%) | | 170 (24.6%) | | 71 (20.9%) | | .221^$^ |  | 78 (25.0%) | 44 (21.7%) | .**045**^&^ |  | 92 (24.3%) | 27 (19.9%) | .352^&^ |
| Female | 789 (76.6%) | | 521 (75.4%) | | 268 (79.1%) | |  |  | 234 (75.0%) | 159 (78.3%) |  |  | 287 (75.7%) | 109 (80.1%) |  |
| **Age, mean (SD)** | 63.6 (9.3) | | 64.0 (8.9) | | 62.9 (9.9) | | .070^$^ |  | 63.9 (9.0) | 63.0 (10.2) | .255^$^ |  | 64.1 (8.8) | 62.89 (9.6) | .159^$^ |
| **BMI, mean (SD)** | 27.8 (4.7) | | 27.7 (4.9) | | 28.0 (4.5) | | .467^$^ |  | 27.7 (4.6) | 28.2 (4.5) | .175^$^ |  | 27.8 (5.1) | 27.6 (4.5) | .664^$^ |
| **Affected joint, n (%)** | | |  | |  | | .164^&^ |  |  |  | .059^&^ |  |  |  | **.004^&^** |
| Knee | 528 (51.3%) | | 368 (53.3%) | | 160 (47.2%) | |  |  | 157 (50.3%) | 107 (52.7%) |  |  | 211 (55.7%) | 53 (39.0%) |  |
| Hip | 232 (22.5%) | | 152 (22.0%) | | 80 (23.6%) | |  |  | 75 (24.0%) | 41 (20.2%) |  |  | 77 (20.3%) | 39 (28.7%) |  |
| Both | 270 (26.2%) | | 171 (24.7%) | | 99 (29.2%) | |  |  | 80 (25.6%) | 55 (27.1%) |  |  | 91 (24.0%) | 44 (32.4%) |  |
| **Artificial joint, n (%)** | | |  | |  | | .392^&^ |  |  |  | .397^&^ |  |  |  | .841^&^ |
| Yes | 130 (12.6%) | | 92 (13.3%) | | 38 (11.2%) | |  |  | 43 (13.8%) | 22 (10.8%) |  |  | 49 (12.9%) | 16 (11.8%) |  |
| **WOMAC, median [IQR]** | | |  | |  | |  |  |  |  |  |  |  |  |  |
| Pain | 2.80 [1.40, 4.47] | | 2.60 [1.20, 4.40] | | 3.20 [1.80, 4.80] | | **<.001^§^** |  | 2.80 [1.40, 4.20] | 3.00 [2.00, 4.40] | **.031^§^** |  | 2.40 [1.00, 4.70] | 3.40 [1.80, 5.25] | **.003^§^** |
| Function | 2.41 [1.12, 4.00] | | 2.12 [0.94, 3.81] | | 2.94 [1.41, 4.44) | | **<.001^§^** |  | 2.31 [1.16, 3.60] | 2.94 [1.35, 4.17] | **.002^§^** |  | 1.94 [0.82, 3.94] | 3.00 [1.47, 4.79] | **.001^§^** |
| **VR-12, mean (SD)** |  | |  | |  | |  |  |  |  |  |  |  |  |  |
| PCS90 | 38.5 (8. 5) | | 39.0 (8.6) | | 37.4 (8.1) | | **.005**^$^ |  | 38.7 (8.2) | 37.8 (8.0) | .247^$^ |  | 39.3 (8.9) | 36.8 (8.1) | **.005^$^** |
| MCS90 | 49.9 (11.0) | | 50.3 (11.0) | | 48.9 (11.1) | | .064^$^ |  | 50.3 (11.1) | 49.5 (11.4) | .228^$^ |  | 50.3 (11.0) | 48.1 (10.5) | **.043^$^** |
| **GSE, mean (SD)** | 3.1 (0.6) | | 3.1 (0.6) | | 3.0 (0.6) | | .138^$^ |  | 3.1 (0.5) | 3.0 (0.6) | .171^$^ |  | 3.1 (0.6) | 3.0 (0.5) | .455^$^ |
| **Ho-PA, mean (SD)** | 2.9 (0.9) | | 2.9 (0.9) | | 3.0 (1.0) | | .061^$^ |  | 2.9 (0.9) | 3.0 (0.9) | .215^$^ |  | 2.9 (0.9) | 3.0 (1.0) | .162^$^ |

HKT: Hip and knee training, CO: control; Standard deviation (SD); Interquartile range (IQR); WOMAC: The mean score of the WOMAC subscales pain and function, with scores ranging from 0 to 10 (best to worst scale). VR-12: Veterans Rand-12 of which the Physical component score (PCS90) and the Mental Component Score (MCS90) was calculated with a value of 50 indicating the mean American Norm 1990 (worst to best). General self-efficacy scale (GSE) with scores ranging from 1-4 (worst to best) and Health-oriented activity status (Ho-AS) with scores ranging from 1 to 5 (best to worst). *percentages add up to 100% within strata of characteristics. Comparison of completers and dropouts: $ = Student’s t-test; & = Chi-squared test; § = Mann-Whitney-U-test.

**Additional Table S7**: Socio-demographic characteristics at baseline of the matched pairs study population (n=1030).

|  | | | | | |  |  |  |
| --- | --- | --- | --- | --- | --- | --- | --- | --- |
|  |  | **HKT**  n = 515 | | **CO**  n = 515 | | | |  |
| Labor situation | employed | 55.1 | % | | 55.2 | | % | |
|  | retired | 34.5 | % | | 32.5 | | % | |
|  | unemployed | 3.0 | % | | 3.2 | | % | |
|  | family members | 7.4 | % | | 9.2 | | % | |
|  | missing N (% n = 515) | 14 | (2.7) | | 13 | | (2.5) | |
| Complexity of work | low | 19.7 | % | | 26.2 | | % | |
|  | moderate | 66.4 | % | | 63.4 | | % | |
|  | high | 12.3 | % | | 5.5 | | % | |
|  | very high | 1.6 | % | | 4.8 | | % | |
|  | missing N (% n = 515) | 393 | (76.3) | | 370 | | (71.8) | |
|  |  |  |  | |  | |  | |
| Years of school education | ≤ 9 years | 39.3 | % | | 33.6 | | % | |
|  | 10 years | 39.3 | % | | 47.7 | | % | |
|  | 13 years (A-Level) | 21.3 | % | | 18.7 | | % | |
|  | missing N (% n = 515) | 426 | (82.7) | | 408 | | (79.2) | |
|  |  |  |  | |  | |  | |
| Level of education | none | 8.7 | % | | 9.2 | | % | |
|  | vocational training | 78.8 | % | | 79.2 | | % | |
|  | Master craftsman/technician | 8.7 | % | | 5.8 | | % | |
|  | Bachelor degree | 1.0 | % | | 1.7 | | % | |
|  | Master degree | 2.9 | % | | 4.2 | | % | |
|  | missing N (% n =515) | 411 | (79.8) | | 395 | | (76.7) | |

HKT: Hip and knee training, CO: control; Mean (M); Standard deviation (SD); Number of missing data (missing N)

| **Parameter** | **WOMAC pain ^$^** | | | |  | **WOMAC function ^$^** | | | |
| --- | --- | --- | --- | --- | --- | --- | --- | --- | --- |
|  | **numDF** | **denDF** | **F-value** | **p** |  | **numDF** | **denDF** | **F-value** | **p** |
| ***PRIMARY ANALYSIS (MI sets)*** | | | | | | | | | |
| *Intercept* | 1 | 1028 | 8263.46 | **< .001** |  | 1 | 1028 | 6861.76 | **< .001** |
| *Time* | 1 | 1028 | 0.20 | .656 |  | 1 | 1028 | 20.66 | **< .001** |
| *Group* | 1 | 1027 | 0.40 | .526 |  | 1 | 1027 | 0.312 | .576 |
| *Ps* | 1 | 1027 | 272.70 | **< .001** |  | 1 | 1027 | 282.70 | **< .001** |
| *Time x Group* | 1 | 1028 | 21.54 | **< .001** |  | 1 | 1028 | 10.54 | **.001** |
| ***SENSITIVITY ANALYSES*** | | | | | | | | | |
| 1. ***All available data (AA)*** | | | | | | | | | |
| *Intercept* | 1 | 1024 | 6937.13 | **< .001** |  | 1 | 1017 | 5678.17 | **< .001** |
| *Time* | 1 | 769 | 30.09 | **< .001** |  | 1 | 772 | 0.01 | .935 |
| *Group* | 1 | 1024 | 0.40 | .527 |  | 1 | 1017 | 0.33 | .566 |
| *Ps* | 1 | 1024 | 285.13 | **< .001** |  | 1 | 1017 | 300.72 | **< .001** |
| *Time x Group* | 1 | 769 | 37.05 | **< .001** |  | 1 | 772 | 15.55 | **< .001** |
| 1. ***Complete Case (CC) ^&^*** | | | | | | | | | |
| *Intercept* | 1 | 756 | 4926.84 | **< .001** |  | 1 | 756 | 4051.47 | **< .001** |
| *Time* | 1 | 756 | 19.33 | **< .001** |  | 1 | 756 | 0.21 | .645 |
| *Group* | 1 | 755 | 2.46 | .117 |  | 1 | 755 | 0.20 | .656 |
| *Ps* | 1 | 755 | 235.17 | **< .001** |  | 1 | 755 | 245.82 | **< .001** |
| *Time x Group* | 1 | 756 | 31.27 | **< .001** |  | 1 | 756 | 12.01 | **< .001** |

**Additional Table S8**: Primary and sensitivity analyses with fixed-effect ANOVA tables of the linear mixed models for WOMAC pain and function (t0 - t3).

$ = log-transformed; & = CC based on the primary endpoint analysis (t0, t3): HKT: n = 357, CO: n = 401. Ps = propensity score.

NumDF = degrees of freedom of numerator; denDF = degreed of freedom of denominator. Level of significance is set at alpha = 0.025 (two-sided, Bonferroni-adjusted).

**Additional Table S9**: Primary and sensitivity analyses with fixed-effect ANOVA tables of the linear mixed models for WOMAC pain and function (t0 - t24).

| **Parameter** | **WOMAC pain ^$^** | | | |  | **WOMAC function ^$^** | | | |
| --- | --- | --- | --- | --- | --- | --- | --- | --- | --- |
|  | **numDF** | **denDF** | **F-value** | **p** |  | **numDF** | **denDF** | **F-value** | **p** |
| ***PRIMARY ANALYSIS (MI sets)*** | | | | | | | | | |
| *Intercept* | 1 | 4112 | 10785.07 | **<.001** |  | 1 | 4112 | 9136.61 | **<.001** |
| *Time* | 4 | 4112 | 0.76 | .552 |  | 4 | 4112 | 10.46 | **<.001** |
| *Group* | 1 | 1027 | 1.75 | .186 |  | 1 | 1027 | 0.162 | .687 |
| *Ps* | 1 | 1027 | 199.43 | **<.001** |  | 1 | 1027 | 201.18 | **<.001** |
| *Time x Group* | 4 | 4112 | 4.88 | **<.001** |  | 4 | 4112 | 3.625 | **.006** |
| ***SENSITIVITY ANALYSES*** | | | | | | | | | |
| 1. ***All available data (AA)*** | | | | | | | | | |
| *Intercept* | 1 | 2837 | 7471.29 | **<.001** |  | 1 | 2890 | 6307.68 | **<.001** |
| *Time* | 4 | 2837 | 11.50 | **<.001** |  | 4 | 2890 | 0.79 | .528 |
| *Group* | 1 | 1027 | 4.21 | .040 |  | 1 | 1023 | 0.90 | .343 |
| *Ps* | 1 | 1027 | 235.40 | **<.001** |  | 1 | 1023 | 247.87 | **<.001** |
| *Time x Group* | 4 | 2837 | 9.09 | **<.001** |  | 4 | 2890 | 7.37 | **<.001** |
| 1. ***Complete Case (CC) ^&^*** | | | | | | | | | |
| *Intercept* | 1 | 1672 | 2695.59 | **<.001** |  | 1 | 1672 | 2286.50 | **<.001** |
| *Time* | 4 | 1672 | 1.69 | .148 |  | 4 | 1672 | 1.88 | .112 |
| *Group* | 1 | 417 | 9.78 | **.002** |  | 1 | 417 | 5.02 | .026 |
| *Ps* | 1 | 417 | 96.79 | **<.001** |  | 1 | 417 | 94.12 | **<.001** |
| *Time x Group* | 4 | 1672 | 3.98 | **<.001** |  | 4 | 1672 | 5.75 | **<.001** |

$ = log-transformed; & = CC based on the follow-up analysis on WOMAC pain and function (t0 – t24): HKT: n = 155, CO: n = 265. Ps = propensity score.

NumDF=degrees of freedom of numerator; denDF=degreed of freedom of denominator. For secondary outcomes, level of significance is set at alpha=0.05.

**Additional Table S10:** Sensitivity analyses for WOMAC pain and function at t3, t6, t12 and t24 months change from baseline (cfb, 95% CI).

|  | **Control (CO)** |  | **Hip Knee Training (HKT)** |  | **Estimated treatment difference CO - HKT** | | **p-value** | **Effect Size** |
| --- | --- | --- | --- | --- | --- | --- | --- | --- |
| ***All available data (AA)*** | | | | | | | |  |
| *Primary Outcomes* |  |  |  |  |  | |  |  |
| WOMAC |  |  |  |  |  | |  |  |
| pain t3 | 0.01 (-0.21; 0.23) |  | -0.57 (-0.79; -0.34) |  | 0.58 (0.35; 0.80) | | **<.001** | 0.27 |
| function t3 | 0.14 (-0.05; 0.32) |  | -0.16 (-0.35; 0.03) |  | 0.30 (0.10; 0.49) | | **.003** | 0.15 |
|  |  |  |  |  |  | |  |  |
| *Secondary Outcomes* |  |  |  |  |  | |  |  |
| WOMAC |  |  |  |  |  | |  |  |
| pain t6 | -0.03 (-0.26; 0.19) |  | -0.55 (-0.79; -0.32) |  | 0.52 (0.29; 0.75) | | **<.001** | 0.24 |
| pain t12 | -0.11 (-0.33; 0.11) |  | -0.62 (-0.85; -0.38) |  | 0.51 (0.27; 0.74) | | **<.001** | 0.24 |
| pain t24 | -0.00 (-0.23; 0.23) |  | -0.50 (-0.74; -0.26) |  | 0.50 (0.26; 0.74) | | **<.001** | 0.23 |
|  |  |  |  |  |  | |  |  |
| function t6 | 0.24 (0.05; 0.43) |  | -0.16 (-0.37; 0.04) |  | 0.40 (0.20; 0.60) | | **<.001** | 0.19 |
| function t12 | 0.21 (0.02; 0.40) |  | -0.25 (-0.45; -0.05) |  | 0.47 (0.27; 0.66) | | **<.001** | 0.23 |
| function t24 | 0.27 (0.08; 0.47) |  | -0.16 (-0.37, 0.05) |  | 0.43 (0.23; 0.64) | | **<.001** | 0.21 |
| ***Complete Case (CC)*** | | | | | | | |  |
| *Primary Outcomes* |  |  |  |  |  |  | |  |
| WOMAC |  |  |  |  |  |  | |  |
| pain t3 | 0.03 (-0.24; 0.30) |  | -0.43 (-0.75; 0.11) |  | 0.46 (0.16; 0.76) | **<.002** | | 0.21 |
| function t3 | 0.16 (-0.06; 0.38) |  | -0.18 (-0.45; 0.10) |  | 0.33 (0.08; 0.59) | **.010** | | 0.16 |
|  |  |  |  |  |  |  | |  |
| *Secondary Outcomes* |  |  |  |  |  |  | |  |
| WOMAC |  |  |  |  |  |  | |  |
| pain t6 | 0.03 (-0.24; 0.30) |  | -0.41 (-0.73; 0.09) |  | 0.44 (0.14; 0.74) | **.003** | | 0.20 |
| pain t12 | 0.03 (-0.24; 0.30) |  | -0.48 (-0.80; 0.16) |  | 0.51 (0.21; 0.81) | **<.001** | | 0.24 |
| pain t24 | 0.08 (-0.20; 0.35) |  | -0.35 (-0.67; 0.03) |  | 0.43 (0.12; 0.73) | .**005** | | 0.20 |
|  |  |  |  |  |  |  | |  |
| function t6 | 0.32 (0.09; 0.55) |  | -0.16 (-0.44; 0.11) |  | 0.49 (0.23; 0.74) | **<.001** | | 0.24 |
| function t12 | 0.31 (0.08; 0.54) |  | -0.25 (-0.53; 0.02) |  | 0.56 (0.31; 0.82) | **<.001** | | 0.27 |
| function t24 | 0.32 (0.10; 0.55) |  | -0.16 (-0.43, 0.12) |  | 0.48 (0.22; 0.74) | **<.001** | | 0.23 |

Linear Mixed Models (Time, Treatment, Time*Treatment, PS); WOMAC (0-10, best to worst): logarithmic estimates back-transformed to original scale. Positive estimated treatment difference indicate benefit for HKT. P-values for time*treatment interaction of the LMMs. Significance in bold is set at alpha=.0025 to account for post-hoc testing with respect to the two primary outcomes. For secondary outcomes alpha = 0.05 without claiming confirmatory interpretation of p-values. Positive effect sizes indicate benefit for HKT versus CO.

**Additional Table S11:** Comparison of estimated treatment difference CO – HKT for primary and sensitivity analyses for WOMAC pain and function at t3, t6, t12 and t24 months change from baseline (cfb, 95% CI).

|  |  |  | **Estimated treatment difference CO - HKT** | | | | | | | | | |  | | |
| --- | --- | --- | --- | --- | --- | --- | --- | --- | --- | --- | --- | --- | --- | --- | --- |
|  | **Primary Analysis (MI)** | | | |  | | **All available data (AA)** | | |  | | **Complete case analysis (CC)** | | | |
|  | **Estimated treatment difference CO - HKT** | | | **Effect**  **Size** | |  | | **Estimated treatment difference CO - HKT** | **Effect**  **Size** | |  | **Estimated treatment difference CO - HKT** | | **Effect**  **Size** |  |
| *Primary Outcomes* |  | | |  | |  | |  |  | |  |  | |  |  |
| WOMAC |  | | |  | |  | |  |  | |  |  | |  |  |
| pain t3 | 0.47 (0.27-0.66) | | | 0.22 | |  | | 0.58 (0.35; 0.80) | 0.27 | |  | 0.46 (0.16; 0.76) | | 0.21 |  |
| function t3 | 0.27 (0.11-0.44) | | | 0.13 | |  | | 0.30 (0.10; 0.49) | 0.15 | |  | 0.33 (0.08; 0.59) | | 0.16 |  |
|  |  | | |  | |  | |  |  | |  |  | |  |  |
| *Secondary Outcomes* |  | | |  | |  | |  |  | |  |  | |  |  |
| WOMAC |  | | |  | |  | |  |  | |  |  | |  |  |
| pain t6 | 0.29 (0.08; 0.51) | | | 0.13 | |  | | 0.52 (0.29; 0.75) | 0.24 | |  | 0.44 (0.14; 0.74) | | 0.20 |  |
| pain t12 | 0.33 (0.12; 0.55) | | | 0.15 | |  | | 0.51 (0.27; 0.74) | 0.24 | |  | 0.51 (0.21; 0.81) | | 0.24 |  |
| pain t24 | 0.34 (0.12; 0.55) | | | 0.16 | |  | | 0.50 (0.26; 0.74) | 0.23 | |  | 0.43 (0.12; 0.73) | | 0.20 |  |
|  |  | | |  | |  | |  |  | |  |  | |  |  |
| function t6 | 0.22 (0.03; 0.41) | | | 0.11 | |  | | 0.40 (0.20; 0.60) | 0.19 | |  | 0.49 (0.23; 0.74) | | 0.24 |  |
| function t12 | 0.33 (0.14; 0.52) | | | 0.16 | |  | | 0.47 (0.27; 0.66) | 0.23 | |  | 0.56 (0.31; 0.82) | | 0.27 |  |
| function t24 | 0.29 (0.09; 0.48) | | | 0.14 | |  | | 0.43 (0.23; 0.64) | 0.21 | |  | 0.48 (0.22; 0.74) | | 0.23 |  |

Estimated treatment difference calculated from Linear Mixed Models (Time, Treatment, Time*Treatment, PS); WOMAC (0-10, best to worst): logarithmic estimates back-transformed to original scale. Positive estimated treatment difference and positive effect sizes indicate benefit for HKT versus CO.

**Additional Information S12:** Exercise adherence and reasons for non-attendance.

Exercise adherence between t0 and t3 was reported by 72% of all HKT participants (n = 369). Thereof, 157 (43%) and 276 (75%) participants attended all scheduled group and home training sessions, respectively. Non-attendance in one or more sessions was reported by 196 (53%, group) and 80 (22%, home) participants. Data with inconsistent reports on attendance and sessions missed were not included in the aforementioned numbers (n = 16 (3%, group sessions) and 13 (3%, home sessions). The most frequently reported reasons for skipping training sessions were “family/work duties” (yes = 149, no = 50), “experiencing pain” (yes = 72, no = 92), “exercises too difficult” (yes = 17, no = 128), “exercise is not enjoyable” (yes = 16, no = 127), “book instructions were not understood” (yes = 4, no = 139), and “public transport problems” (yes = 4, no = 141). Additional self-defined reasons for skipping training sessions included illness, hospital or inpatient rehabilitation (n = 30), exercise-related complaints (n = 5), vacation (n = 8), musculoskeletal complaints (n = 9), motivational issues (n = 5), and others (n = 13).

**Additional Table S13:** Frequency of self-reported exercise-related pain during HKT (t3), n (%).

|  |  | |  | |  | | |  | |  | | |  | |  |  |  |  |
| --- | --- | --- | --- | --- | --- | --- | --- | --- | --- | --- | --- | --- | --- | --- | --- | --- | --- | --- |
| Incidence | | Frequency | | | | Duration | | | | | | Intensity ^§^ | | | | | | |
| Yes | 190 (36.9) | Always | | 9 (1.7) | | | During | | 54 (10.5) | | 1-2 | | | 33 (6.4%) | | | |  |
| No | 157 (30.5) | Most of the time | | 38 (7.4) | | | At same day | | 39 (7.6) | | 3-5 | | | 136 (26.4%) | | | |  |
|  |  | Sometimes | | 110 (21.4) | | | Until next day | | 74 (14.4) | | 6-8 | | | 38 (7.4%) | | | |  |
|  |  | Rarely | | 51 (9.9) | | | Longer | | 28 (5.2) | | 9-10 | | | 6 (1.2%) | | | |  |
| Missing data | 168 (32.6) | Missing data | | 307 (59.6) | | | Missing data | | 321 (62.3) | | Missing data | | | 302 (58.6%) | | | |  |
|  |  | |  | |  | | | WOMAC Pain (t0) | |  | | |  | | | |  | |

^§^10-point Likert scale with *1 = weak pain* and *10 = extreme pain*.

**Additional Table S14:** Concomitant care related to hip and knee training during the previous follow-up period, n (% of n = 515/group).

|  | |  | | |  | | |  | | |  | | |  | | | |  | | |  | | |  | | |  | | |  | | |
| --- | --- | --- | --- | --- | --- | --- | --- | --- | --- | --- | --- | --- | --- | --- | --- | --- | --- | --- | --- | --- | --- | --- | --- | --- | --- | --- | --- | --- | --- | --- | --- | --- |
|  | No training | | | | | | AOK-HKT (group training) | | | | | | AOK-HKT (home-based training) | | | | AOK machine-based training | | | | | | Other hip/knee training | | | | | Number of responders | | | | |
|  | HKT | | | CO | | | HKT | | | CO | | | HKT | | CO | | HKT | | | CO | | | HKT | | | CO | | HKT | | | CO | |
| t3 | n. a. | | | 211 (41.0) | | | n. a. | | | 13 (2.5) | | | n. a. | | 0 (0.0) | | n. a. | | | 7 (1.4) | | | n. a. | | | 165 (32.0) | | n. a. | | | 396 (76.9) | |
| t6 | 97 (18.8) | | | 207 (40.2) | | | 64 (12.4) | | | 6 (1.2) | | | 70 (13.6) | | 8 (1.6) | | 10 (1.9) | | | 8 (1.6) | | | 110 (21.4) | | | 149 (28.9) | | *307 (59.6) | | | *378 (73.4) | |
| t12 | 111 (21.6) | | | 182 (35.3) | | | 21 (4.1) | | | 9 (1.7) | | | 17 (3.3) | | 0 (0.0) | | 2 (0.4) | | | 5 (1.0) | | | 76 (14.8) | | | 152 (29.5) | | 227 (44.1) | | | 348 (67.6) | |
| t24 | 108 (21.0) | | | 173 (33.6) | | | 43 (8.3) | | | 21 (4.1) | | | 31 (6.0) | | 13 (2.5) | | 15 (2.9) | | | 16 (3.1) | | | 139 (27.0) | | | 164 (31.8) | | *291 (56.5) | | | *360 (69.9) | |
|  |  | | |  | | |  | | |  | | |  | |  | |  | | |  | | |  | | |  | |  | | |  | |
|  | | |  | | |  | | |  | | |  | | | |  | | | WOMAC Pain (t0) | | |  | | |  | | | |  | | |  |

* does not correspond to the row total, because of multiple entries (only t6, t24)

**Additional Table S15:** Concomitant care related to other health care offers of the AOK-BW and else during the previous follow-up period, n (% of n = 515/group).

|  | |  | | |  | | |  | | |  | | |  | | |  | | |  | | |  | | |  | | |  |  |
| --- | --- | --- | --- | --- | --- | --- | --- | --- | --- | --- | --- | --- | --- | --- | --- | --- | --- | --- | --- | --- | --- | --- | --- | --- | --- | --- | --- | --- | --- | --- |
|  | No | | | | | | Yes | | | | | | Specified 1^st^ | | | | | | Specified 2^nd^ | | | | | | Specified 3^rd^ | | | | | |
|  | HKT | | | CO | | | HKT | | | CO | | | HKT | | | CO | | | HKT | | | CO | | | HKT | | | CO | | |
| t3 | 244 (47.4) | | | 362 (70.3) | | | 123 (23.9) | | | 48 (9.3) | | | 121 (23.5) | | | 58 (11.3) | | | 29 (5.6) | | | 18 (3.5) | | | 8 (1.6) | | | 9 (1.7) | | |
| t6 | 225 (43.7) | | | 356 (69.1) | | | 88 (17.1) | | | 43 (8.3) | | | 86 (16.7) | | | 56 (10.9) | | | 27 (5.2) | | | 13 (2.5) | | | 8 (1.6) | | | 4 (0.8) | | |
| t12 | 202 (39.2) | | | 344 (66.8) | | | 105 (20.4) | | | 45 (8.7) | | | 109 (21.2) | | | 50 (9.7) | | | 43 (8.3) | | | 10 (1.9) | | | 6 (1.2) | | | 3 (0.6) | | |
| t24 | 206 (40.0) | | | 321 (62.3) | | | 101 (19.6) | | | 46 (8.9) | | | 103 (20.0) | | | 53 (10.3) | | | 36 (7.0) | | | 13 (2.5) | | | 10 (1.9) | | | 4 (0.8) | | |
|  |  | | |  | | |  | | |  | | |  | | |  | | |  | | |  | | |  | | |  | | |
|  | | |  | | |  | | |  | | |  | | |  | | |  | | |  | | |  | | |  | | |  |

Participants were asked to specify up to three health care offers (Specified 1^st to^ 3^rd^). A large variety of different health care were mentioned that were mostly related to physical exercise (i. e. “stay fit”, mind-body exercises, stretching, aqua training, back strengthening exercises, etc). Some others were related to other lifestyle interventions such as nutrition or healthy weight.

**Additional Table S16:** Baseline characteristics of complete case population (t0, t3) of HKT and subgroup CO-exercise (CO participants having reported to engage in hip/knee joint-specific exercises between t0 and t3).

|  | **Hip and Knee Training (HKT)**  n = 357 | | **Control (CO)**  n = 178 | **P-value** | **Test** |
| --- | --- | --- | --- | --- | --- |
| Women (n, %) | 265 (74.2) | | 137 (77.0) |  |  |
| Age (years), mean (SD) | 63.36 (9.22) | | 62.83 (8.52) | 0.526 |  |
| Body Mass Index (kg/m2), mean (SD) | 27.99 (4.79) | | 27.75 (5.41) | 0.603 |  |
| OA lifetime prevalence, (n, %) |  | |  |  |  |
| Knee | 183 (52.0) | | 93 (53.1) |  |  |
| Hip | 86 (24.4) | | 38 (21.7) |  |  |
| Both | 83 (23.6) | | 44 (25.1) |  |  |
| Joint replacement (hip/knee), (n, %) | 48 (13.6) | | 28 (16.2) | 0.509 |  |
| WOMAC, Median (IQR) |  |  | |  |  |
| Pain | 2.60 (1.40, 4.20) | | 2.60 (1.20, 4.35) | 0.811 | nonnorm |
| Function | 2.35 (1.06, 3.65) | | 2.03 (0.78, 3.59) | 0.224 | nonnorm |
| VR-12, Mean (SD) |  | |  |  |  |
| PCS90 | 38.92 (8.29) | | 39.47 (8.86) | 0.475 |  |
| MCS90 | 50.88 (10.75) | | 50.71 (11.26) | 0.864 |  |
| General self-efficacy scale (GSE), Mean (SD) | 3.11 (0.55) | | 3.10 (0.55) | 0.842 |  |
| Health-oriented activity status (Ho-AS), Mean (SD) | 2.88 (0.87) | | 2.59 (0.78) | <0.001 |  |

**Legend table S16:** HKT: Hip and knee training, CO: control; Interquartile range (IQR); WOMAC: The mean score of the WOMAC subscales pain and function, with scores ranging from 0 to 10 (best to worst scale). VR-12: Veterans Rand-12 of which the Physical component score (PCS90) and the Mental Component Score (MCS90) was calculated with a value of 50 indicating the mean American Norm 1990 (worst to best). General self-efficacy scale (GSE) with scores ranging from 1-4 (worst to best) and Health-oriented activity status (Ho-AS) with scores ranging from 1 to 5 (best to worst).

**Additional Table S17:** Within-group estimates of change from baseline (cfb, 95% CI) and the according between-group estimated treatment differences (ETDs) at t3 for WOMAC pain and function of HKT and subgroup CO-exercise (CO participants having reported to engage in hip/knee joint-specific exercises between t0 and t3).

|  | **Control (CO)**  **n = 515** | |  | | **Hip Knee Training (HKT)**  **n = 515** | |  | | **Estimated treatment difference (ETD)**  **CO - HKT** | | **p-value** | | **Effect Size** | |
| --- | --- | --- | --- | --- | --- | --- | --- | --- | --- | --- | --- | --- | --- | --- |
| *Primary Outcomes* |  | |  | |  | |  | |  | |  | |  | |
| WOMAC |  | |  | |  | |  | |  | |  | |  | |
| pain t3 | -0.12 (-0.34; 0.11) | |  | | -0.54 (-0.70; -0.38) | |  | | 0.42 (0.15; 0.69) | | **< 0.001** | | 0.20 | |
| function t3 | 0.18 (0.03; 0.33) | |  | | -0.12 (-0.24; -0.01) | |  | | 0.30 (0.11; 0.50) | | **= 0.001** | | 0.15 | |
|  | |  | |  | |  | |  | |  | |  |  |  |

**Legend table S17:** Linear Mixed Models (Time, Treatment, Time*Treatment, Propensity Score (PS)); WOMAC (0-10, best to worst): logarithmic estimates back-transformed to original scale; Significance in bold is set at alpha=.0025 to account for post-hoc testing with respect to the two primary outcomes. Positive effect sizes indicate benefit for HKT versus CO.

1. MM: Motor learning and mobilization | S: Strengthening | H: Hip specific (numbers correspond to exercise book) | K: Knee specific (numbers correspond to additional exercise leaflet) [↑](#footnote-ref-1)
2. M-Plane (Movement plane): S: Sagittal plane | F: Coronal plane | T: Transverse plane [↑](#footnote-ref-2)
3. RoI: Region of interest [↑](#footnote-ref-3)
4. EB: Exercise balls | RB: Elastic rubber bands | WC: Weight cuff [↑](#footnote-ref-4)
5. MM: Motor learning and mobilization | S: Strengthening | H: Hip specific (numbers correspond to exercise book) | K: Knee specific (numbers correspond to additional exercise leaflet) [↑](#footnote-ref-5)
6. M-Plane (Movement plane): S: Sagittal plane | F: Coronal plane | T: Transverse plane [↑](#footnote-ref-6)
7. RoI: Region of interest [↑](#footnote-ref-7)
8. EB: Exercise balls | RB: Elastic rubber bands | WC: Weight cuff [↑](#footnote-ref-8)
9. MM: Motor learning and mobilization | S: Strengthening | H: Hip specific (numbers correspond to exercise book) | K: Knee specific (numbers correspond to additional exercise leaflet) [↑](#footnote-ref-9)
10. M-Plane (Movement plane): S: Sagittal plane | F: Coronal plane | T: Transverse plane [↑](#footnote-ref-10)
11. RoI: Region of interest [↑](#footnote-ref-11)
12. EB: Exercise balls | RB: Elastic rubber bands | WC: Weight cuff [↑](#footnote-ref-12)
13. MM: Motor learning and mobilization | S: Strengthening | H: Hip specific (numbers correspond to exercise book) | K: Knee specific (numbers correspond to additional exercise leaflet) [↑](#footnote-ref-13)
14. M-Plane (Movement plane): S: Sagittal plane | F: Coronal plane | T: Transverse plane [↑](#footnote-ref-14)
15. RoI: Region of interest [↑](#footnote-ref-15)
16. EB: Exercise balls | RB: Elastic rubber bands | WC: Weight cuff [↑](#footnote-ref-16)
