# Supplementary material for: Effectiveness of an 11-week exercise intervention for patients with hip or knee osteoarthritis: results of a quasi-experimental pragmatic trial
Source: BMC Sports Sci Med Rehabil. 2024 Jan 20;16:24. doi: 10.1186/s13102-023-00779-0 (PMC10799538; doi:10.1186/s13102-023-00779-0)
Supplement: Supplementary file 3 — Additional File 3. 2017 CONSORT checklist of information to include when reporting a randomized trial assessing nonpharmacologic treatments (NPTs)*. Modifications of the extension appear in italics and blue. [file 13102_2023_779_MOESM3_ESM.docx]

2017 CONSORT checklist of information to include when reporting a randomized trial assessing nonpharmacologic treatments (NPTs)*. Modifications of the extension appear in italics and blue.

| Section/Topic Item | Checklist item no. | CONSORT item | *Extension for NPT trials* | Location |
| --- | --- | --- | --- | --- |
| Title and abstract |  |  |  |  |
|  | 1a | Identification as a randomized trial in the title |  | p.1 |
|  | 1b | Structured summary of trial design, methods, results, and conclusions (for specific guidance see CONSORT for abstracts) | *Refer to CONSORT extension for abstracts for NPT trials* | p. 2-3 |
| Introduction |  |  |  |  |
| Background and objectives | 2a | Scientific background and explanation of rationale |  | p. 4 |
|  | 2b | Specific objectives or hypotheses |  | pp. 4-5 |
| Methods |  |  |  |  |
| Trial design | 3a | Description of trial design (such as parallel, factorial) including allocation ratio | *~~When applicable, how care providers were allocated to each trial group~~* | p. 5  *Extension N/A* |
|  | 3b | Important changes to methods after trial commencement (such as eligibility criteria), with reasons |  | Suppl. File 1 |
| Participants | 4a | Eligibility criteria for participants | When applicable, eligibility criteria for centers and for *care providers* | p. 5-6  Suppl. File 2  *p. 5, 9* |
|  | 4b | Settings and locations where the data were collected |  | p. 5-6 |
| Interventions*†* | 5 | The interventions for each group with sufficient details to allow replication, including how and when they were actually administered | *Precise details of both the experimental treatment and comparator* | see CERT Checklist |
| Outcomes | 6a | Completely defined pre-specified primary and secondary outcome measures, including how and when they were assessed |  | p. 9 ff. |
|  | 6b | Any changes to trial outcomes after the trial commenced, with reasons |  | Suppl. File 1 |
| Sample size | 7a | How sample size was determined | *When applicable, details of whether and how the clustering by care providers or centers was addressed* | pp. 12  Krauss et al. BMC Public Health 2016; 16: 367  *Suppl. File 1* |
|  | 7b | When applicable, explanation of any interim analyses and stopping guidelines |  | Suppl. File 1 |
| Randomization: |  |  |  |  |
| - Sequence generation | 8a-10 | Method used to generate the random allocation sequence |  | N/A (quasi-experimental trial), matching process outlined p. 13 |
|  |  |  |  |  |
| Blinding | 11a | If done, who was blinded after assignment to interventions (for example, participants, care providers, those assessing outcomes) and how | *~~Whether or not those administering co-interventions were blinded to group assignment~~* | p. 12  Suppl. File 1 |
|  | 11b | If relevant, description of the similarity of interventions | *~~If blinded, method of blinding and description of the similarity of interventions~~ N. A.* | N/A  *N/A* |
|  | 11c |  | *~~If blinding was not possible, description of any attempts to limit bias.~~ No attempts done.* | *Not reported* |
| Statistical methods | 12a | Statistical methods used to compare groups for primary and secondary outcomes | *When applicable, details of whether and how the clustering by care providers or centers was addressed.* | pp. 13  *Suppl. File 1* |
|  | 12b | Methods for additional analyses, such as subgroup analyses and adjusted analyses |  | pp. 14-15  Suppl. File 1 |
| Results |  |  |  |  |
| Participant flow (a diagram is strongly recommended) | 13a | For each group, the numbers of participants who were randomly assigned, received intended treatment, and were analyzed for the primary outcome | *The number of care providers or centers performing the intervention in each group and the number of patients treated by each care provider or in each center* | Figure 1  pp. 15-16  *p. 20*  *Suppl. File 1* |
|  | 13b | For each group, losses and exclusions after randomization, together with reasons |  | Figure 1  p. 16  Suppl. File 7 |
|  | 13c |  | *For each group, the delay between randomization and the initiation of the intervention* | *p. 15*  *No further details available* |
|  | new |  | *Details of the experimental treatment and comparator as they were implemented.* | *p. 20, 24* |
| Recruitment | 14a | Dates defining the periods of recruitment and follow-up |  | p. 15 |
|  | 14b | Why the trial ended or was stopped |  | p. 15 |
| Baseline data | 15 | A table showing baseline demographic and clinical characteristics for each group | *~~When applicable, a description of care providers (case volume, qualification, expertise, etc.) and centers (volume) in each group.~~*  *Information is not available for this trial* | (Suppl. File 6)  Table 3  p. 16  Suppl. File 8 |
| Numbers analyzed | 16 | For each group, number of participants (denominator) included in each analysis and whether the analysis was by original assigned groups |  | Figure 1  Table 4  Suppl. File 9, 10 (Footnotes) |
| Outcomes and estimation | 17a | For each primary and secondary outcome, results for each group, and the estimated effect size and its precision (such as 95% confidence interval) |  | pp. 12-17  Table 4,  Figure 2a/2b  Figure 3  Figure 4  Suppl. File 9  Suppl. File 10c |
|  | 17b | ~~For binary outcomes, presentation of both absolute and relative effect sizes is recommended.~~ N. a.: no binary outcomes and concomitant care was not analyzed in a statistical manner. |  | N/A |
| Ancillary analyses | 18 | Results of any other analyses performed, including subgroup analyses and adjusted analyses, distinguishing pre-specified from exploratory |  | p. 18-20  Suppl. File 1  Suppl. File 9  Suppl. File 10a  Suppl. File 10b  Suppl. File 10c |
| Harms | 19 | All important harms or unintended effects in each group (for specific guidance see CONSORT for harms) |  | p. 19  Suppl. File 12 |
| **Discussion** |  |  |  |  |
| Limitations | 20 | Trial limitations, addressing sources of potential bias, imprecision, and, if relevant, multiplicity of analyses | *In addition, take into account the choice of the comparator, lack of or partial blinding, ~~and unequal expertise of care providers or centers in each group~~ not known* | P. 24-25  Suppl. File 1 |
| Generalizability | 21 | Generalizability (external validity, applicability) of the trial findings | *Generalizability (external validity) of the trial findings according to the intervention, comparators, patients, and care providers and centers involved in the trial* | p. 25 |
| Interpretation | 22 | Interpretation consistent with results, balancing benefits and harms, and considering other relevant evidence |  | p. 20 ff.  p. 25-26 |
| Other information |  |  |  |  |
| Registration | 23 | Registration number and name of trial registry |  | p. 3 |
| Protocol | 24 | Where the full trial protocol can be accessed, if available |  | p. 5 |
| Funding | 25 | Sources of funding and other support (such as supply of drugs), role of funders |  | p. 28 |

**Additions or modifications to the 2010 CONSORT checklist. CONSORT = Consolidated Standards of Reporting Trials*

*†The items 5, 5a, 5b, 5c, 5d are consistent with the Template for Intervention Description and Replication (TIDieR) checklist*
